# Characterization of proanthocyanidin metabolism in pea (*Pisum sativum*) seeds

**DOI:** 10.1186/s12870-014-0238-y

**Published:** 2014-09-16

**Authors:** Kiva Ferraro, Alena L Jin, Trinh-Don Nguyen, Dennis M Reinecke, Jocelyn A Ozga, Dae-Kyun Ro

**Affiliations:** Department of Biological Sciences, University of Calgary, 2500 University Dr. NW, Calgary, Alberta Canada; Plant BioSystems, Department of Agricultural, Food, and Nutritional Science, University of Alberta, Edmonton, Alberta Canada

**Keywords:** Proanthocyanidin, Pea seeds, *Pisum sativum*, Anthocyanidin reductase, Flavan-*3*-ols, Flavonoid biosynthesis, Leucoanthocyanidin reductase

## Abstract

**Background:**

Proanthocyanidins (PAs) accumulate in the seeds, fruits and leaves of various plant species including the seed coats of pea (*Pisum sativum*), an important food crop. PAs have been implicated in human health, but molecular and biochemical characterization of pea PA biosynthesis has not been established to date, and detailed pea PA chemical composition has not been extensively studied.

**Results:**

PAs were localized to the ground parenchyma and epidermal cells of pea seed coats. Chemical analyses of PAs from seeds of three pea cultivars demonstrated cultivar variation in PA composition. ‘Courier’ and ‘Solido’ PAs were primarily prodelphinidin-types, whereas the PAs from ‘LAN3017’ were mainly the procyanidin-type. The mean degree of polymerization of ‘LAN3017’ PAs was also higher than those from ‘Courier’ and ‘Solido’. Next-generation sequencing of ‘Courier’ seed coat cDNA produced a seed coat-specific transcriptome. Three cDNAs encoding anthocyanidin reductase (*PsANR*), leucoanthocyanidin reductase (*PsLAR*), and dihydroflavonol reductase (*PsDFR*) were isolated. *PsANR* and *PsLAR* transcripts were most abundant earlier in seed coat development. This was followed by maximum PA accumulation in the seed coat. Recombinant PsANR enzyme efficiently synthesized all three *cis*-flavan-*3*-ols (gallocatechin, catechin, and afzalechin) with satisfactory kinetic properties. The synthesis rate of *trans-*flavan-*3*-ol by co-incubation of PsLAR and PsDFR was comparable to *cis*-flavan-*3*-ol synthesis rate by PsANR. Despite the competent PsLAR activity *in vitro*, expression of *PsLAR* driven by the *Arabidopsis ANR* promoter in wild-type and *anr* knock-out *Arabidopsis* backgrounds did not result in PA synthesis.

**Conclusion:**

Significant variation in seed coat PA composition was found within the pea cultivars, making pea an ideal system to explore PA biosynthesis. *PsANR* and *PsLAR* transcript profiles, PA localization, and PA accumulation patterns suggest that a pool of PA subunits are produced in specific seed coat cells early in development to be used as substrates for polymerization into PAs. Biochemically competent recombinant PsANR and PsLAR activities were consistent with the pea seed coat PA profile composed of both *cis-* and *trans-*flavan-*3*-ols. Since the expression of *PsLAR* in *Arabidopsis* did not alter the PA subunit profile (which is only comprised of *cis*-flavan-*3*-ols), it necessitates further investigation of *in planta* metabolic flux through PsLAR.

**Electronic supplementary material:**

The online version of this article (doi:10.1186/s12870-014-0238-y) contains supplementary material, which is available to authorized users.

## Background

*Pisum sativum* (pea) seeds are a rich source of minerals, proteins, starch and antioxidants. Dry pea seeds are widely used in agriculture as feed for livestock and are gaining interest as feed in aquaculture. Pea seeds, one of the oldest grain legumes consumed by humans, are also gaining wide recognition as a healthy food ingredient in the human diet due to the low glycemic index of the starches [1].

**Figure 1:**
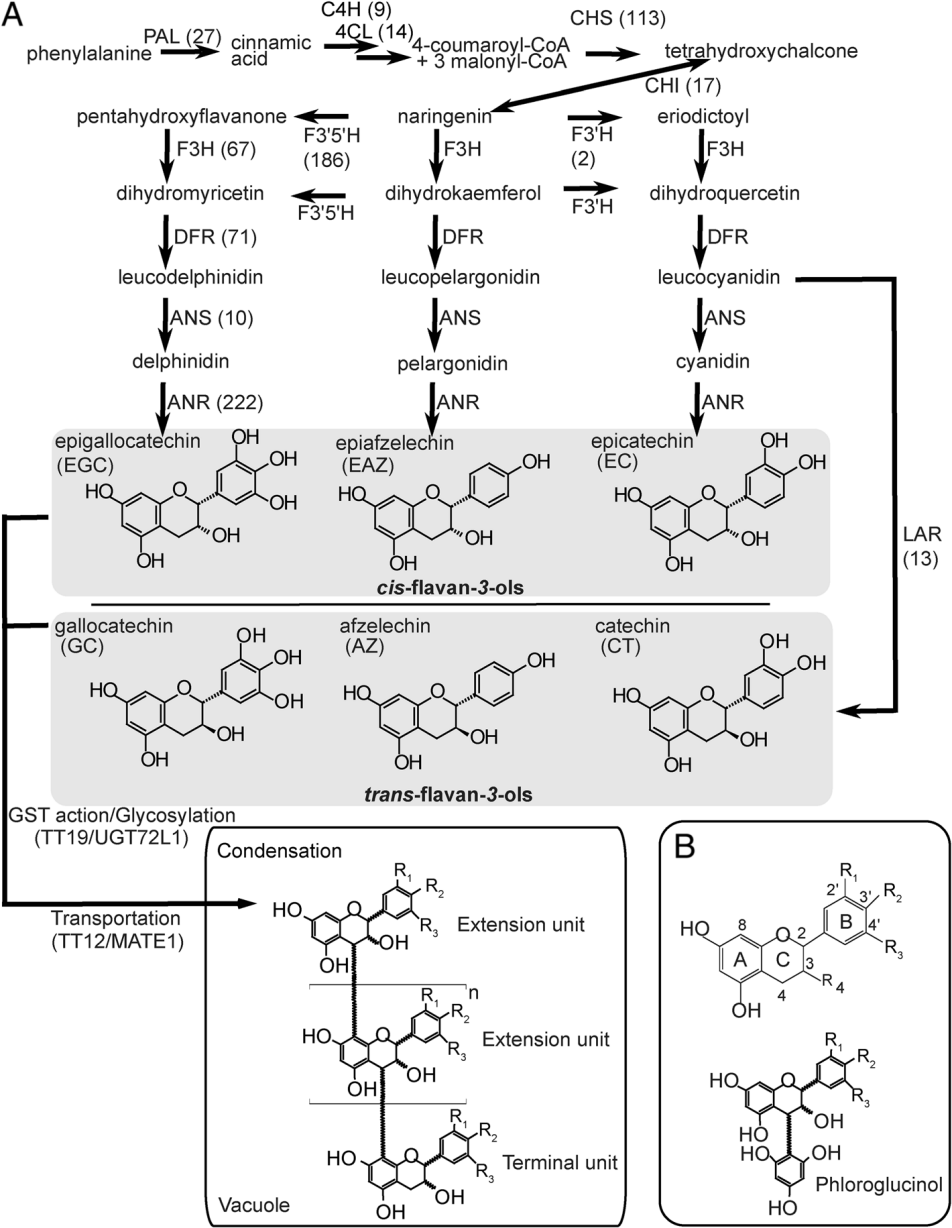
**Proanthocyanidin biosynthetic pathway with transcript levels of each biosynthetic gene estimated by 454 read numbers, and structures of proanthocyanidins and their derivatized products. A)** Proanthocyanidin biosynthetic pathway. PAL, phenylalanine ammonia lyase; C4H, cinnamate 4-hydroxylase; 4CL, 4-coumarate:CoA ligase; CHS, chalcone synthase; CHI, chalcone isomerase; F3’5’H, flavonoid 3’5’-hydroxylase; F3’H, flavonoid 3’-hydroxylase; F3H, flavanone 3-hydroxylase; DFR, dihydroflavonal 4-reductase; ANS, anthocyanidin synthase; ANR, anthocyanidin reductase; LAR, leucoanthocyanidin reductase. Values in brackets indicate the read numbers from 454-pyrosequencing. **B)** C4-C8 linkage in PA-phloroglucinol adduct structures.

Flavonoids are of particular interest due to their strong antioxidant properties. Proanthocyanidins (PAs; Figure 1), also known as condensed tannins, are a subclass of flavonoids that accumulate in seed coats of a number of plant species including pea, and are thought to function as protective agents against biotic and abiotic stresses [2]. Historically, PAs were considered as anti-nutritional compounds in pulse nutritional studies because they can precipitate proteins and reduce bioavailability of some minerals. However, recent research suggests that PAs have considerable potential for use as a novel therapy or treatment for a range of human health conditions, including cardiovascular disease, cancer establishment and progression, and bacterial infections [3]. The use of PAs as a plant-based health-beneficial component in the human diet has led to renewed interest in this class of flavonoids in food crops [4,5]. Specifically, studies indicate that PA polymer length is inversely related to bioavailability in humans [6]. Therefore, identification of variation in PA composition and length within *Pisum sativum,* as well as the mechanisms responsible for this variation would be a great benefit for breeding new cultivars with additional health beneficial properties.

PAs are derived from the flavonoid branch of the phenylpropanoid pathway (Figure 1). Chemical diversity can be introduced early in the pathway by regio-selective cytochrome P450 enzymes, F3′H and F3′5′H (Figure 1, see legend for full names), which hydroxylate 3′- or 3′,5′-positions of naringenin B-ring [7,8]. Two consecutive reactions by F3H [9] and DFR then synthesize colorless flavan-3,4-diols (leucoanthocyanidins) [10], which are further converted to (-)-*cis*-flavan-*3*-ols through the sequential reactions of anthocyanidin synthase (ANS) [11] and anthocyanidin reductase (ANR) [12] or to (+)-*trans*-flavan-*3*-ols by leucoanthocyanidin reductase (LAR) [13].

Biosynthesis of these flavan-*3*-ol monomers is believed to occur on the cytosolic surface of the endoplasmic reticulum, yet PAs themselves accumulate in the vacuole [14,15]. Two multi-drug and toxic compound extrusion (MATE) transporters, TT12 and MATE1, characterized from *Arabidopsis thaliana* and *Medicago truncatula*, respectively, are able to transport epicatechin-3-*O*-glucoside (glycosylated *cis*-flavan-*3*-ol) across the tonoplastic membrane, but they were not able to transport aglycones (i.e., cyanidin and epicatechin) [16,17]. Therefore, glycosylation of flavan-*3*-ols appears to be necessary for the MATE-mediated transport, which is further supported by the recent discovery of an epicatechin-specific glycosyltransferase from *M. truncatula* [18]. Mechanistically, the MATE transporters are flavonoid H^+^-anti-porters, and the proton gradient required for this H^+^-anti-porter is believed to be generated by AHA10 (a H^+^-ATPase) on the tonoplast membrane [19].

In contrast to the transporter-mediated delivery of PA monomers, vesicle-mediated transport has also been proposed *in planta. Arabidopsis* mutant *tt19*, which encodes a glutathione-S-transferase-like protein, accumulates PA derivatives including flavan-*3*-ols in small vacuole-like structures [20]. TT19 may itself bind flavonoids to protect them from oxidation in the cytosol rather than conjugate glutathione to the flavan-*3*-ols [21]. A Golgi-independent vesicle-mediated trafficking pathway has also been proposed for anthocyanins, a group of pigments closely related to flavan-*3*-ols [22]. Recently, vesicles containing PA were identified and named as tannosome from grape (*Vitis vinifera*) and several other vascular plants [23]. This result also supports the implication of vesicle-mediated trafficking, but the vesicles appear to be derived from chloroplasts, which is in contrast to the ER/cytosolic biosynthesis of PA and hence requires further investigation.

PA polymers consist of flavan-*3*-ol aglycone subunits, suggesting a β-glucosidase within the vacuole may be required. Alternatively, deglycosylation may be coupled with condensation, which itself remains unknown. PA polymer length, composition of subunits, and C-C bond stereochemistry varies between plant species, suggesting enzymatic control of condensation [3]. Laccases and peroxidases have been considered as potential condensing enzymes, although to date no PA condensing enzyme has been identified. One candidate, TT10, a putative laccase-like polyphenol oxidase, was proposed, but this enzyme appears to function in the apoplastic space where it converts colourless extractable PAs into their brown non-extractable oxidized form [24]. However, TT10 recombinant enzyme can oxidize epicatechin (EC), resulting in the formation of oligomers, although the resulting *in vitro* interflavan linkages are not naturally occurring [24]. It is possible that a protein partner, such as the dirigent protein involved in lignin coupling [25], is necessary for proper PA oligomerization, but non-enzymatic polymerization has not yet been ruled out [3].

Much of the research on seed coat-derived PAs has been conducted using the non-crop species *Arabidopsis* and *M. truncatula*. However, both of these species produce PA polymers composed almost exclusively of the *cis*-flavan-*3*-ol, epicatechin (Figure 1) [14,26]. Pea offers unique advantages to study PA biosynthesis. Pea seeds are substantially larger than those of *Arabidopsis* and *M. truncatula*, allowing for ready isolation of the seed coat tissue, the primary site of PA accumulation [27]. Also, a long history of agricultural breeding of pea has produced a wide variety of pea cultivars. Thousands of accessions of pea (*Pisum sativum*) exist around the world, providing both a rich source of genetic diversity and nutritional variation [28]. It is likely that variations in PA composition and polymer length exist in pea, and this could provide valuable resources to improve desirable PAs by breeding or biotechnological means. Despite the importance of pea as a crop and the possible value in understanding pea PA metabolism, comprehensive chemical and biochemical studies of PAs in pea have not been achieved to date. As the first step to advance the knowledge of PA biosynthesis in pea, we histologically localized PAs, determined PA accumulation, and chemically characterized the PAs of three PA-accumulating cultivars within the pea seed coat over development. The transcript abundance of two key PA branch point genes, *PsANR* and *PsLAR,* were profiled over development, and the enzymes they encode were biochemically characterized. Using these data, we developed a working hypothesis of PA biosynthesis in pea seed coat tissue.

## Results

### Localization of PAs in developing ‘Courier’ pea seed coats

**Figure 2:**
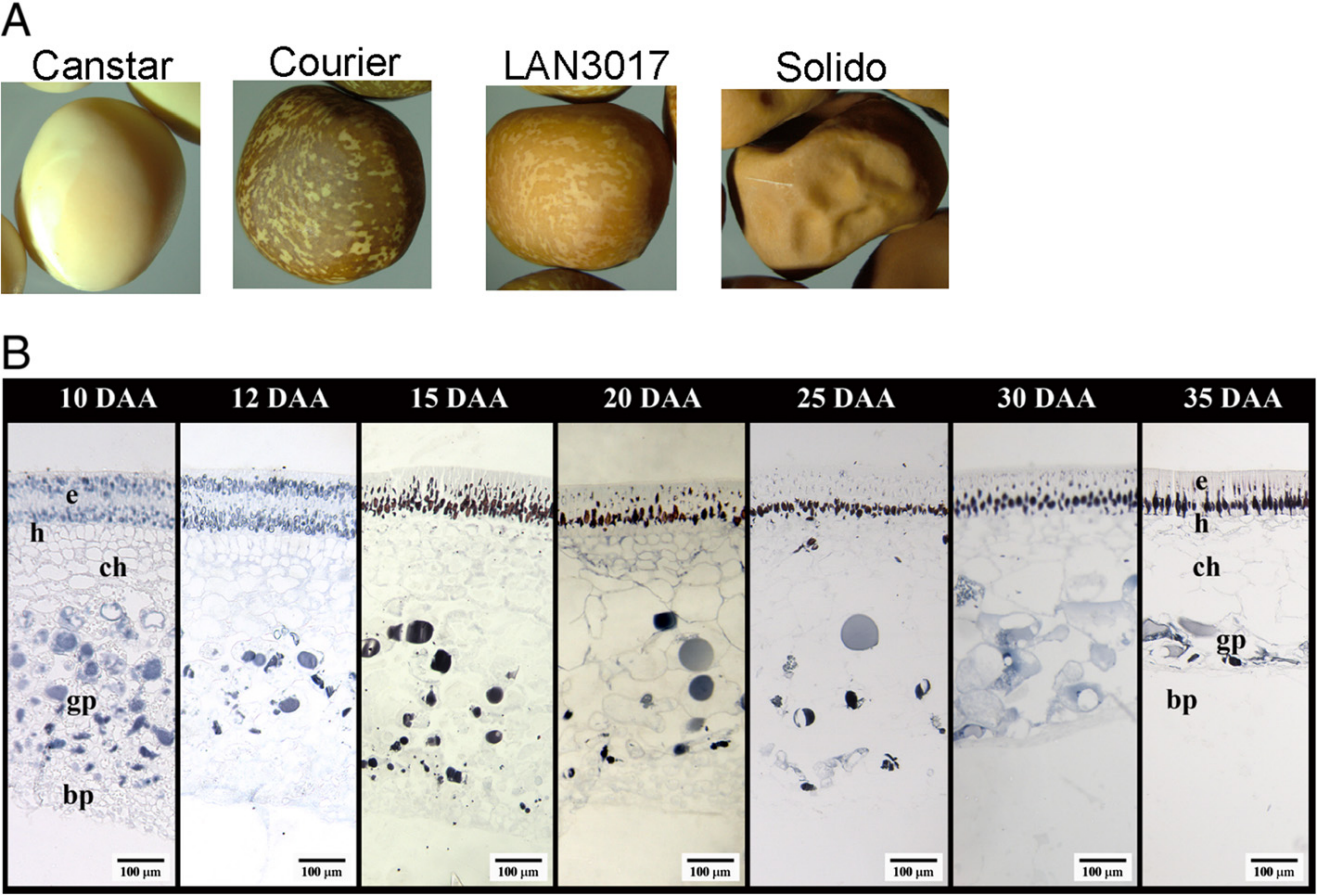
**Pea seeds and PA localization in developing pea seed coat. A)** Representative images of pea seeds from each cultivar. **B)** Cotyledon mid-region cross sections of ‘Courier’ pea seed coats. e, epidermal layer; h, hypodermal layer; ch, chlorenchyma layer; gp, ground parenchyma layer; bp, branched parenchyma layer. DAA: days after anthesis.

PAs were localized in the pea seed coats of ‘Courier’ (Figure 2) over development using a *p*-dimethylaminocinnamaldehyde (DMACA) staining method [29]. PAs mainly accumulated intracellularly (likely the vacuole) in the cells of the epidermal and ground parenchyma layers of the seed coat throughout development (Figure 2). As the seed matured, the cells of the epidermal layer of the seed coat sclerified, and the intercellular space and vacuolar size decreased. As a result, the vacuolar-localized PAs are visualized in the inner side of the epidermal layer. Also note that the inner seed coat cell layers are progressively crushed by the expanding embryo as the seed develops (after 15 DAA; Figure 2).

### Proanthocyanidin profile of *Pisum sativum* cultivars

**Figure 3:**
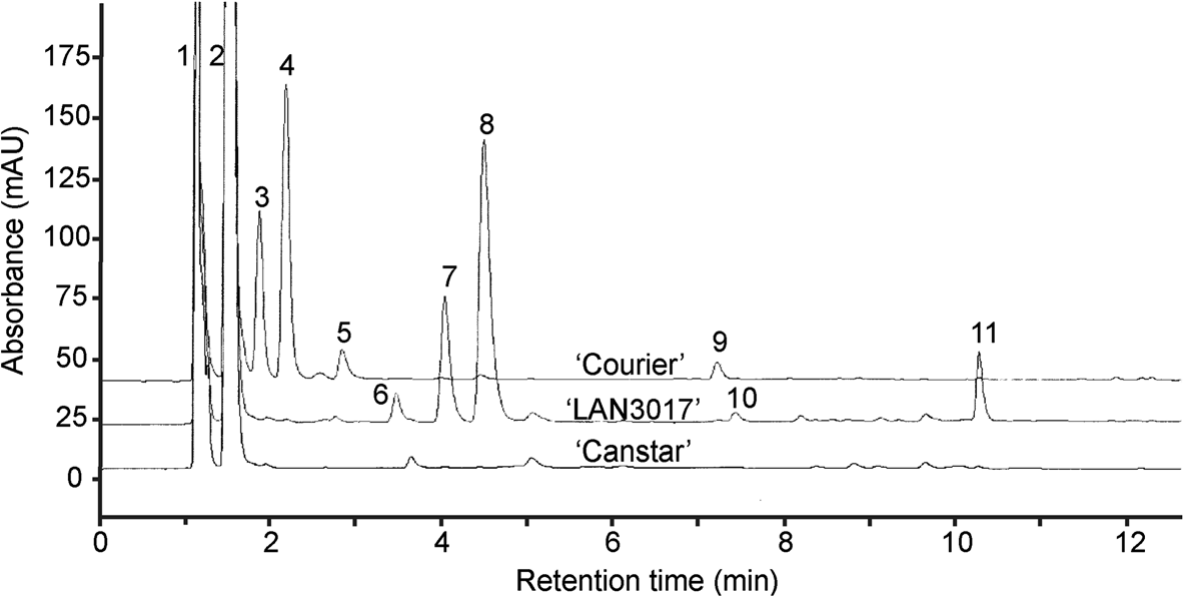
**HPLC chromatograms of the phloroglucinol acid hydrolysis products from pea seeds of ‘Courier’, ‘LAN3017’, and ‘Canstar’.** 1. L-Ascorbic acid; 2. Phloroglucinol; 3. Gallocatechin-(4α-2)-phloroglucinol; 4. Epigallocatechin-(4β-2)-phloroglucinol; 5. Gallocatechin; 6. Putative Catechin-(4β-2)-phloroglucinol; 7. Catechin-(4α-2)-phloroglucinol; 8. Epicatechin-(4β-2)-phloroglucinol; 9. Epigallocatechin; 10. Catechin; 11. Epicatechin.

The PA content and subunit composition of the seed coats from three PA-accumulating pea cultivars (Courier, LAN3017, and Solido) and a cultivar containing minimal PAs (Canstar) were determined by acid-catalyzed cleavage followed by phloroglucinol derivatization (phloroglucinolysis) (Table 1 and Figure 3) [30]. This method allows the determination of PA subunit composition and concentration by the comparison of the retention properties of reaction products with those of flavan-*3*-ol standards and other well characterized PA phloroglucinol reaction products. Flavan-*3*-ol PA extension units form phloroglucinol adducts at their C4 position while terminal flavan-*3*-ol units are released as flavan-*3*-ol monomers (Figure 1), the ratio of which allows determination of the mean degree of polymerization (mDP).

**Table 1:** **PA chemical analyses of ‘Courier’, ‘Solido’, and ‘LAN3017’ pea seeds and seed coats**

**PA analysis using phloroglucinolysis and RP-HPLC-DAD in mature pea seeds**				
**Peak ID**	**Compound**	**‘Courier’**	**‘Solido’**	**‘LAN3017’**
3	GC-P	29.23 ± 0.73^a^	28.99 ± 0.88	1.36 ± 0.02
4	EGC-P	55.38 ± 1.05	51.16 ± 1.27	0.79 ± 0.05
5	GC	9.88 ± 0.29	10.53 ± 0.14	nd^b^
6	CT-P isomer	nd	nd	4.98 ± 0.00
7	CT-P	nd	0.22 ± 0.01	21.41 ± 0.01
8	EC-P	0.37 ± 0.01	0.60 ± 0.02	65.37 ± 0.03
9	EGC	5.13 ± 0.04	8.24 ± 0.21	nd
10	CT	nd	nd	0.94 ± 0.00
11	EC	nd	0.27 ± 0.02	5.15 ± 0.02
mDP		6.7 ± 0.2	5.3 ± 0.1	16.4 ± 0.0
Conversion yield^c^		83.9 ± 1.6	78.3 ± 4.9	59.1 ± 0.9
Total seed PA^d^		416.0 ± 7.7	264.1 ± 14.6	96.7 ± 13.2
Butanol-HCl quantification of PA content from pea seed coats				
Total seed coat PA (%)^e^		4.57 ± 0.03	4.51 ± 0.09	5.10 ± 0.07

^a^Molar % ± SE (n = 2); ^b^nd, not detected; ^c^Yield of PA extract calculated.

^d^Total seed PA content based on characterized PA subunits, expressed as mg/100 g dry weight of whole seeds. GC-P, gallocatechin-(4α → 2)-phloroglucinol; GC, gallocatechin; EGC-P, epigallocatechin-(4β → 2)-phloroglucinol; EGC, epigallocatechin; CT-P, catechin-(4α → 2)-phloroglucinol; CT, catechin; EC-P, epicatechin-(4β → 2)-phloroglucinol; EC, epicatechin.

^e^Total seed coat PA content expressed as % = mg/100 mg dry weight of seed coat sample using 80% methanol extraction. Proanthocyanidin extract from ‘CDC Acer’ pea seed coats purified as described by Jin et al. [41] was used as a standard for the butanol-HCl assay. Data are means ± SE (n = 3).

No PA subunits were detected in the RP-HPLC chromatography of ‘Canstar’ seed extracts (’Canstar’ has clear-coloured seed coats, and the yellowish colouration of the seed is from the cotyledons; Figure 2A and 3). PA subunits were detected in the seed extracts of cultivars ‘Courier’, ‘Solido’, and ‘Lan3017’, that have brown or brown-speckled seed coats (Table 1; Figures 2A and 3). In the seeds of pea cultivars ‘Courier’ and ‘Solido’, similar PA flavan-*3*-ol extension and terminal unit profiles were detected (Table 1). The PA flavan-*3*-ol extension units were nearly exclusively prodelphinidin (2′,3′,4′-hydroxylated flavan-*3*-ols), where epigallocatechin (EGC; peak 4, Figure 3; Table 1) was the most abundant flavan-*3*-ol extension subunit followed by gallocatechin (GC; peak 3, Figure 3; Table 1). The PA terminal subunits of these pea cultivars mainly consisted of GC (peak 5) and EGC (peak 9, Figure 3; Table 1). A minimal amount of epicatechin (EC) also occurred in the PA extension subunits (peak 8) in these two cultivars, and in the terminal subunits (peak 11) of ‘Solido’ (Figure 3; Table 1). On the other hand, the PA flavan-*3*-ol extension and terminal subunit profile of ‘LAN3017’ seeds was markedly different from those of ‘Solido’ and ‘Courier’ (Figure 3; Table 1). ‘LAN3017’ contained nearly exclusively procyanidin (2′,3′-hydroxylated flavan-*3*-ols) moieties in the PA polymers, with the majority of the PA extension subunits consisting of EC (peak 8, Figure 3) followed by catechin (CT; peak 7, Figure 3; Table 1).

The mDP of the PA polymers was similar in ‘Courier’ and ‘Solido’ at 5–7 subunits in length. However, the PA mDP was 2 to 3 times greater in ‘LAN3017’ than that in the other pea cultivars (Table 1). The PA extension and terminal subunits in the PA-containing pea cultivars are assumed to be linked in a B-type configuration (C4-C8 or C4-C6) (Figure 1A; C4-C8), as the PA interflavonoid bonds were readily cleaved under the acidic conditions. The identities of the PA subunits detected in the HPLC analysis were further substantiated by LC-MS/MS (Additional file 1: Table S1).

Similar PA levels were found among the PA-containing pea cultivars when the total extractable PA content of the seed coat was estimated using the butanol-HCl method (Table 1). The total extractable PA yield from whole seed extracts was also calculated using the PA extract yield values and the conversion yield of PAs to known subunits with data from the phloroglucinolysis method (Table 1) [31]. The lower PA content values obtained in the whole seed extracts compared to the seed coat extracts are the result of: 1) PA localization in the seed coat and not the embryo of the seeds for all cultivars, and 2) a larger ratio of embryo to seed coat tissue in the seeds of ‘Solido’, and decreased solubility of the longer PA polymers of ‘LAN3017’ in the extraction solvent used in the phloroglucinolysis procedure compared to the shorter PA polymers present in ‘Courier’ and ‘Solido’. Therefore, the total extractable PA content of the seed coat as estimated using the butanol-HCl assay is the method of choice for determining PA content difference among these cultivars.

**Figure 4:**
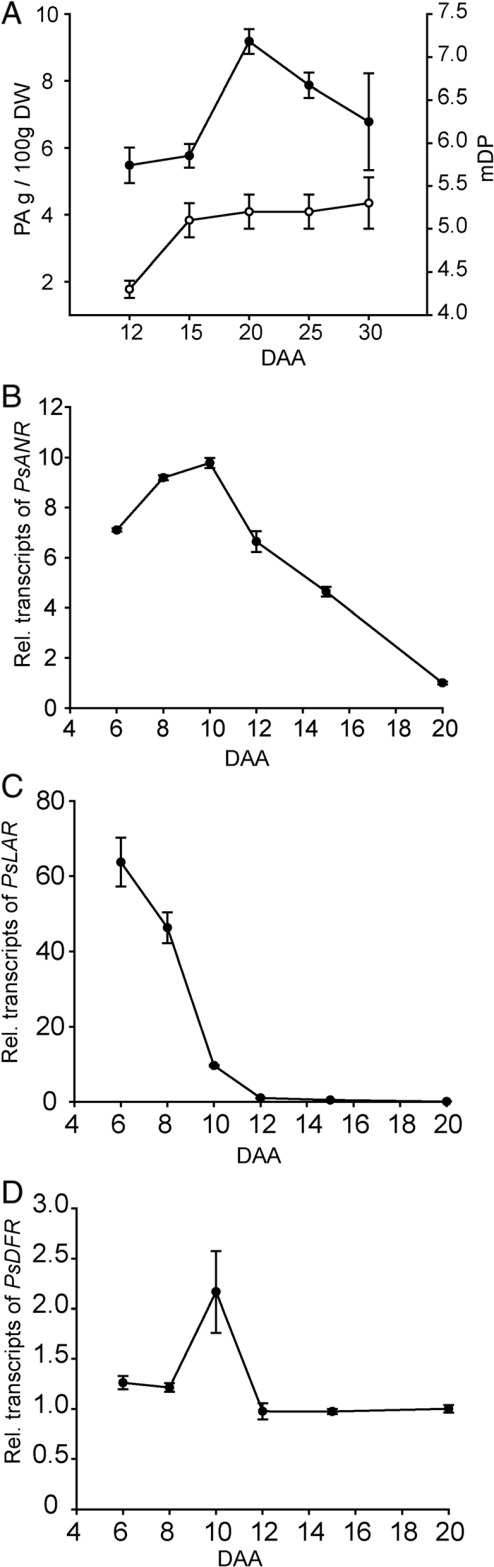
**Temporal profiles of** ***PsANR*** **,** ***PsDFR*** **and** ***PsLAR*** **transcript abundance, PA content and mean degree of polymerization in pea seed coats of ‘Courier’. A)** PA content (black circles) and mean degree of polymerization (mDP: white circles) in developing ‘Courier’ seed coats from 12 to 30 DAA; data are means ± SE (n=3). Relative transcript abundance of ‘Courier’ **B)** *PsANR* **C)** *PsDFR* and **D)** *PsLAR* from 6 to 20 DAA using qRT-PCR. Transcript abundance values of *PsANR* and *PsDFR* were normalized to the 20 DAA, and PsLAR to the 12 DAA samples. Actin was used as the reference gene in all experiments. Data are means ± SE (*PsLAR* and *PsANR*, n = 4; *PsDFR* n = 3).

To further understand PA accumulation in the pea seed coat, the content and composition of ‘Courier’ extractable PAs over development were examined. The molar percent of GC in the extension units increased as seed development progressed, while a small decrease in EGC occurred (Table 2). The mDP of PAs from young seed coats at 12 days after anthesis (DAA) was less than five, then it increased slightly (about one subunit in length) by 15 DAA and it remained at this level until 30 DAA (Figure 4A). At seed maturity, the mDP increased to approximately seven (Table 1). The extractable seed coat PA content increased during development, reaching a maximum level at 20 DAA (Figure 4A). After 20 DAA, the extractable PA content steadily decreased until seed maturation.

**Table 2:** **PA profiles in developing seed coats of ‘Courier’**

	**GC-P**	**EGC-P**	**EC-P**	**GC**	**EGC**	**EC**
**12 DAA** ^**a**^	20.5 ± 1.9^b^	55.8 ± 1.9	0.42 ± 0.02	17.3 ± 0.4	5.65 ± 0.26	0.38 ± 0.03
**15 DAA**	24.8 ± 0.7	55.2 ± 0.1	0.38 ± 0.03	15.0 ± 0.3	4.35 ± 0.36	0.32 ± 0.10
**20 DAA**	27.5 ± 0.6	52.9 ± 0.7	0.36 ± 0.04	14.4 ± 0.7	4.47 ± 0.19	0.29 ± 0.07
**25 DAA**	29.2 ± 0.6	51.6 ± 1.0	0.22 ± 0.19	14.3 ± 0.4	4.51 ± 0.16	0.21 ± 0.01
**30 DAA**	30.8 ± 1.7	49.8 ± 2.2	0.31 ± 0.04	14.1 ± 0.4	4.76 ± 0.21	0.19 ± 0.05

^a^DAA, days after anthesis; ^b^Molar% ± SE (n = 3).

GC-P, gallocatechin-(4α → 2)-phloroglucinol; EGC-P, epigallocatechin-(4β → 2)-phloroglucinol; EC-P, epicatechin-(4β → 2)-phloroglucinol; GC, gallocatechin; EGC, epigallocatechin; EC, epicatechin.

PA content was determined using the phloroglucinolysis and RP-HPLC-DAD analysis method.

### Cloning and characterization *P. sativum* ANR

Pea seed coat PA subunits consisted of a high quantity of *trans*-flavan-*3*-ols (GC and CT) in addition to common *cis*-flavan-*3*-ols (EGC and EC; Table 1). In contrast, the PAs of the closely related legume species *Medicago truncatula* and the model plant *Arabidopsis thaliana* are reported to not contain *trans*-flavan-*3*-ol subunits. These results imply that both LAR and ANR, the key enzymes responsible for the biosynthesis of PA precursors, are highly active in the pea seed coat PA biosynthesis pathway (Figure 1). No biochemical studies of these two key branch enzymes have been conducted in the crop species pea, and thus we pursued thorough biochemical studies of ANR and LAR.

‘Courier’ was chosen as the source of a *PsANR* clone as this cultivar displayed high seed coat PA accumulation as well as significant quantities of *cis*-flavan-*3*-ol PA subunits (Table 1). A full-length *PsANR* clone was retrieved from ‘Courier’ seed coat cDNA using degenerate PCR, followed by rapid amplification of cDNA ends (5’-, 3’-RACE). *PsANR* encodes a 1,017-bp ORF and shares 84% and 60% amino acid identity with *M. truncatula* ANR and *Arabidopsis* ANR, respectively. PsANR is highly conserved among ‘Courier’, ‘LAN3017’ and ‘Solido’, differing by only a single amino acid in ‘LAN3017’ (position 28, Gln to Glu) and in ‘Solido’ (position 327, Ile to Val).

**Figure 5:**
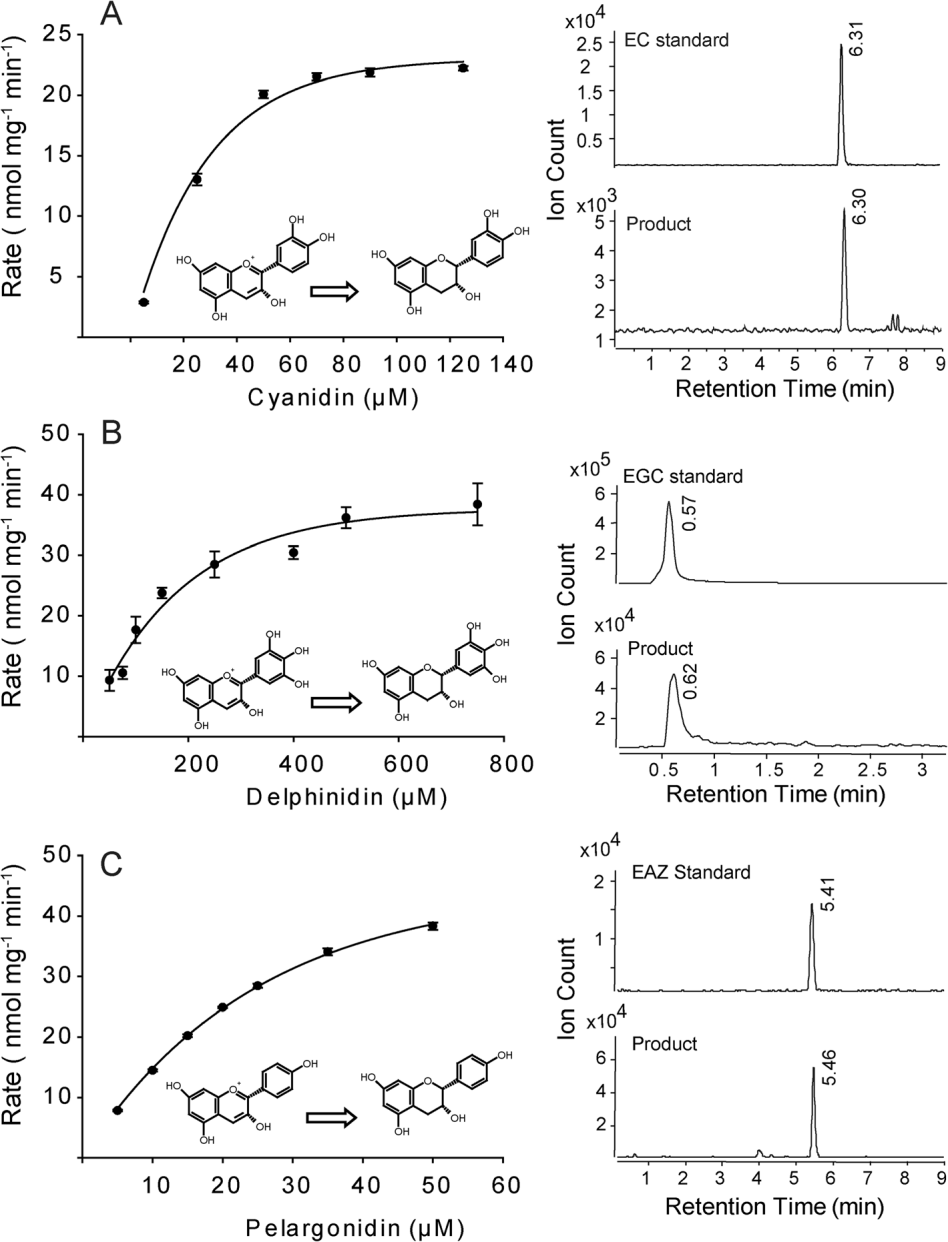
***In vitro*** **characterization of PsANR recombinant enzyme.** **A-C**: PsANR reaction kinetics were explored using cyanidin **(A)**, delphinidin **(B)** and pelargonidin **(C)**. Left: Michaelis-Menten kinetics plots. Each data point represents means ± SE (n = 3). Right: LC-MS identification in reference to authentic standards [(−)-epicatechin (*m/z* = 291), (−)-epigallocatechin (*m/z* = 307), and (−)-epiafzelechin (*m/z* = 275)].

To examine the catalytic activity of PsANR, *PsANR* was expressed as an N-terminal six-histidine tagged recombinant protein and purified using a Ni-NTA column. Based on the pea PA subunit composition data, the primary *in planta* substrate for ‘Courier’ PsANR is expected to be the 2′,3′,4′-hydroxylated anthocyanidin, delphinidin (Figure 1). Therefore, delphinidin as well as two related compounds, 2′,3′-hydroxylated cyanidin and 3′-hydroxylated pelargonidin, were assessed as substrates for recombinant PsANR (Figure 5). When the PsANR enzymatic products were analyzed by LC-MS/MS, they showed identical co-chromatographic and MS/MS patterns with the corresponding authentic *cis*-flavan-*3*-ol standards, EGC, EC and EAZ (Figure 5 and Additional file 2: Figure S1). No flavan-*3*-ol product was detected when NADPH was omitted or if the protein was boiled prior to the assay (data not shown). These results showed that all three compounds can be efficiently used as substrates to produce *cis*-flavan-*3*-ols, and that non-enzymatic conversion of *cis*-flavan-*3*-ols to *trans*-flavan-*3*-ols did not occur under our *in vitro* assay conditions. The optimal pH (using citrate/phosphate and Tris–HCl buffers) and temperature for PsANR activity were determined to be 7.0 and 40°C, respectively. In the optimized reaction condition, the kinetics properties of PsANR for the three substrates were further determined (Figure 5 and Table 3). The rates of the respective product formation (i.e., *cis*-flavan-*3*-ol) from substrates fit well to the Michaelis-Menten kinetics model with minor variations in affinity and turnover number. PsANR showed comparable *k*_cat_ values for all three substrates ranging from 0.5 to 1.2 × 10^−3^ sec^−1^. However, the *K*_*m*_ values for pelagonidin and cyanidin as substrates were approximately 5-fold lower than for delphinidin, making the overall kinetic efficiency of PsANR for delphinidin 2–7 fold lower than for pelagonidin and cyanidin. Interestingly, it was recently reported that ANRs from *Vitis vinifera* (grape) and *Camellia sinensis* (tea) have an intrinsic epimerase activity, producing *trans*-flavan-*3*-ols *in vitro* as well as *cis*-flavan-*3*-ols [32,33]. Of interest to this study is the possibility that ANR could contribute to the formation of *trans*-flavan-*3*-ols, along with its known ability to form *cis*-flavan-*3*-ols. However, we observed no evidence for PsANR epimerase activity for the conversion of *cis*-flavan-*3*-ols to *trans*-flavan-*3*-ols, as *trans*-flavan-*3*-ol products were not observed using *cis*-flavan-*3*-ols (EC and EGC) as substrates in the PsANR recombinant enzyme assays (Additional file 3: Figure S2).

**Table 3:** **PsANR reaction kinetics using cyanidin, pelargonidin or delphinidin as a substrate**

**Substrate**	**K**_**m**_ **(**μ**M)**	**V**_**max**_ **(nmol mg** ^**−1**^ **min** ^**−1**^ **)**	***k***_***cat***_ **(sec** ^**−1**^ **)**	***k***_***cat***_ **/K**_**m**_ **(M** ^**−1**^ **sec** ^**−1**^ **)**
Pelargonidin	39.0 ± 0.1^a^	72.5 ± 2.2^a^	1.2 × 10^−3^	30.7
Cyanidin	37.0 ± 0.2	30.2 ± 5.0	0.5 × 10^−3^	13.5
Delphinidin	183.6 ± 0.2	47.3 ± 6.4	0.8 × 10^−3^	4.3

^a^Data are means ± SE (n = 3).

### Transcriptome of *P. sativum* seed coat

Although ANR activity could be evaluated using commercial substrates, LAR substrates (leucoanthocyanidins; Figure 1) are not stable or commercially available. Due to the lack of substrate, LAR activity was examined using enzymatically synthesized substrates by the DFR recombinant enzyme. However, both *PsDFR* and *PsLAR* clones were not present in the publicly available EST database. During the progress of this work, two Transcript Shotgun Assembly (TSA) data from garden and field pea were released to the NCBI using next-generation sequencings (NGS, Roche/454 sequencing platform) [34,35]. In these data sets, a full length *PsDFR* could be identified, but a *PsLAR* clone was still missing since the seed coat was not included in these sequencing samples.

To improve the current pea TSA data and also to facilitate the present studies of PA metabolism, pea seed coats were physically isolated and pooled from ripening fruits between 10 and 25 DAA. A small scale NGS (a quarter plate) was performed using the Roche/454 sequencing method. Accordingly, a total of 40,903 reads with an average of 392-bp read length were generated, and these individual reads were assembled through MIRA algorithm to yield 16,272 unigenes (5,766 contigs and 10,506 singletons) [36]. These unigenes were annotated by BLASTx against TAIR and UniProt protein sets through the FIESTA bioinformatics pipeline (Plant Biotechnology Institute, Canada). With an E-value of 10^−2^ cut-off, the unigenes showed 9,702 and 9,420 hits against TAIR and UniProt protein sets.

Annotated unigenes were ranked by their abundance, according to the number of reads constituting the contigs (Additional file 4: Table S2). The transcripts among the top 20 highly expressed genes included 1-aminocyclopropane-1-carboxylate oxidase (ethylene biosynthesis; ranked 2nd), indole-3-acetic acid amido synthetase (auxin sequestering, ranked 3rd), methionine synthase (ethylene biosynthetic precursor; rank 4th), and gibberellin 2β-dioxygenase (*PsGA2ox1*, gibberellin deactivation gene; ranked 16th). These results are consistent with gene expression changes observed in other studies (increase in *PsGA2ox1* in the pea seed coat during a similar stage of development [37] and other hormonal regulation of seed development processes [38]). It should be noted that two unigenes annotated as *ANR* and *F3′5′ hydroxylase* were ranked as the 5^th^ and 6^th^ most abundant contigs in the database, indicating that PA biosynthesis is a major metabolic route in pea seed coat.

Next, we assessed the coverage of PA metabolic genes represented in our seed coat-specific TSA data set. The protein sequences of the characterized enzymes involved in PA biosynthesis were curated from *Arabidopsis*, *Medicago sativa* (alfalfa), *M. truncatula*, and petunia (*Petunia* spp.), and were used as BLASTx queries. The identified contigs and singletons with high E-value hits were manually inspected to determine the numbers of reads for each gene. This quantitative analysis revealed that all 12 genes for PA biosynthesis are present in the pea TSA data set, but their read numbers varied significantly (from 2 to 222 out of ~40,000 total reads; Figure 1, numbers in parenthesis). In agreement with the PA chemical phenotype of ‘Courier’ (mostly 3′,4′,5′-hydroxy flavan-*3*-ols), *F3′5′H* showed an abundant read number (186 reads) of transcripts while *F3′H* had only two reads. As these two enzymes compete for the common substrate naringenin, this relative transcript abundance explains the delphinidin-derived PA subunits in ‘Courier’. In this TSA data set, *DFR* and *ANR* were represented by 71 and 222 reads, respectively, and they were present as full-length genes. However, *LAR* had only 13 reads and was present as a partial clone (Figure 1).

### Cloning and characterization *P. sativum* LAR

The deduced protein sequences from the full-length *PsDFR* (1,029-bp ORF) is approximately 38.4 kDa, and it shows 89% and 70% amino acid identity to *M. truncatula* and *Arabidopsis* DFR, respectively. Contigs representing *PsLAR* lacked a portion of the 5’-sequence, and hence the full-length *PsLAR* (1,056-bp ORF) was recovered by 5’-RACE. The encoded PsLAR protein sequence, calculated to be approximately 38.8 kDa, is 85% and 67% identical to *M. truncatula* LAR and *Desmodium uncinatum* LAR, respectively. The LAR characteristic amino acid motifs RFLP, ICCN, and THD were conserved in the PsLAR protein sequence (Additional file 5: Figure S3) [39].

**Figure 6:**
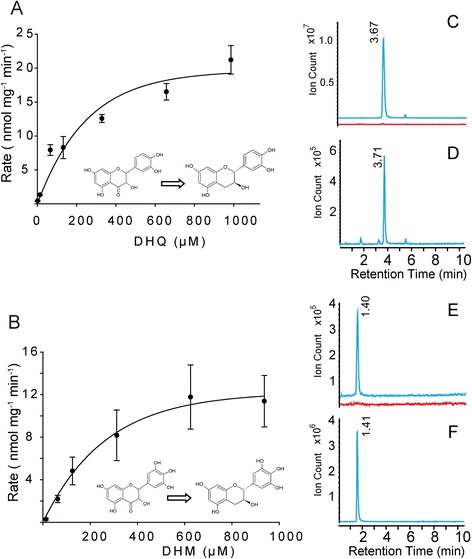
***In vitro*** **PsDFR and PsLAR coupled assays.** Product synthesis rates from the coupled assays were measured using DFR substrates, dihydroquercetin **(A)** and dihydromyricetin **(B)**. Left: pseudo-kinetics plots were inferred from the coupled assays. Each data point represents means ± SE (n = 3). C-F: LC-MS [M + H]^+^ extracted ion chromatographs (**C** and **D** *m/z* = 291; **E** and **F**, *m/z* = 307) of authentic (+)-catechin **(D)** and (+)-gallocatechin **(F)** along with *in vitro* assay products **(C and E)** from PsDFR only (red line) or PsDFR + PsLAR coupled assays (blue line).

To examine their catalytic activities, *PsLAR* and *PsDFR* were expressed as recombinant proteins with N-terminal six-histidine tags and purified using the same method as for PsANR (Additional file 6: Figure S4). Purified PsDFR recombinant enzyme was used to provide PsLAR substrate *in vitro*. In these coupled assays, purified PsDFR and PsLAR were mixed at a 2:1 molar ratio, and the DFR substrate, dihydroquercetin (DHQ) or dihydromyricetin (DHM), was added to the reaction assays in optimized reaction conditions (40°C and a slightly acidic pH of 6). The formation of predicted *trans*-flavan-*3*-ol (CT or GC) was then analyzed by LC-MS in comparison to the authentic standards (Figure 6). Only co-incubation of PsDFR and PsLAR could synthesize compounds displaying [M + H]^+^ ion for CT (*m/z* = 291) or GC (*m/z* = 307) (Figure 6C and E).

Overall, the coupled assays showed very efficient conversions of the substrates, DHQ and DHM. When the coupled assays were performed at 65 μM substrate, 36% conversion of DHQ to CT and 12% conversion of DHM to GC were observed. Despite the inaccuracy to calculate kinetic properties from the coupled assays, PsLAR kinetic values were inferred by plotting product formation rate in relation to varying substrate (DHM and DHQ) concentrations. In these pseudo-kinetic analyses, the product synthesis rates (*V*_*max*_) of the coupled assays were 2 to 3-fold lower than those from ANR but still comparable (Figure 6 and Table 3).

As observed for PsANR activity, the substrate with a lower degree of B-ring hydroxylation (DHQ) was converted more efficiently, even though DHM is the expected native substrate in ‘Courier’ seed coats. For DFR, LAR and ANR, the degree of B-ring hydroxylation of the available substrate is determined by the upstream activity of flavonoid 3’-hydroxylase (F3’H) and flavonoid 3’5’-hydroxylase (F3’5’H) (Figure 1). Preliminary gene expression data from our lab (unpublished) indicates that *PsF3’H* is highly expressed in ‘LAN3017’ versus F3’5’H in ‘Courier’ and ‘Solido’, which matches the observed PA profiles (Table 1). Thus, substrate availability is controlled independently from the substrate preference of these enzymes. The confirmation of an active PsLAR protein when coupled to PsDFR *in vitro*, therefore, supports the abundance of 2,3-*trans*-flavan-*3*-ols found in the pea seed coats.

### Developmental regulation of *PsANR* and *PsLAR* in ‘Courier’ seed coats

With the demonstration of enzymatically competent PsANR and PsLAR, we assumed that coordinated expression of *PsANR* and *PsLAR* determines the PA content and composition in pea seed coats of ‘Courier’. To understand developmental regulation of these two key genes, temporal expression of *PsANR* and *PsLAR* from 6 to 20 DAA in ‘Courier’ seed coat was determined by quantitative real-time PCR (qRT-PCR; Figure 4B and C). The transcript abundance of both *PsANR* and *PsLAR* was high during the earlier stages of pea seed coat development. Both genes displayed a decline in expression as the seed coat matured, but PsLAR transcripts decreased significantly faster than *PsANR* transcript. Seed coat *PsDFR* transcript levels (codes for the enzyme responsible for the production of substrate used by LAR and ANS) were stable from 6 to 20 DAA, except for a 2-fold increase at 10 DAA (Figure 4D). Maximal PA accumulation in ‘Courier’ seed coat did not immediately follow transcriptional induction of *PsANR* and *PsLAR*, but it reached its highest level at 20 DAA (Figure 4A). PA mDP increased to five by 15 DAA, and it remained at this level to 20 DAA (Figure 4A).

### Heterologous expression of *PsLAR* in *Arabidopsis*

*Arabidopsis* lacks *LAR* and does not synthesize *trans*-flavan-*3*-ols. In *Arabidopsis*, all leucoanthocyanidins are channelled to *cis*-flavan-*3*-ols by ANS and ANR. In order to examine if the expression of *PsLAR* in *Arabidopsis* seed coat can re-direct the metabolic flux to *trans*-flavan-*3*-ols, *PsLAR* was expressed with a FLAG-epitope tag by a ~1.3-Kb fragment of *Arabidopsis ANR* promoter [27]. This construct (*P*_*ANR*_*-PsLAR*) was transformed to wild-type *Arabidopsis* as well as *ANR* knock-out (*anr*) *Arabidopsis* mutants identified from a T-DNA knock-out database. We hypothesized that the wild-type *Arabidopsis* expressing *PsLAR* would synthesize PAs comprised of a mixture of *cis*- and *trans*-flavan-*3*-ols while the *Arabidopsis anr* mutant expressing *PsLAR* would produce PAs exclusively composed of *trans*-flavan-*3*-ols.

Using transgenic plants, T-DNA insertions were confirmed by PCR-screening of genomic DNA in the *anr* mutant (data not shown). Subsequently, presence of *PsLAR* transcript and its recombinant enzyme were confirmed by RT-PCR and immunoblot analysis using anti-FLAG antibodies (Additional file 7: Figure S5A/B). Furthermore, activity of the PsLAR was confirmed using crude protein extracted from siliques with and without supplementary recombinant PsDFR (Additional file 7: Figure S5C). Therefore, the transgenic *Arabidopsis* produced functional LAR. Subsequently, the *Arabidopsis* transgenic lines were examined for alteration of PA subunit chemical phenotypes. The *anr* mutant expressing *PsLAR* was used to test for restoration of seed coat color and to detect the presence of DMACA reactive products; the seeds from wild-type *Arabidopsis* expressing *PsLAR* were used to profile monomer units of PA after phloroglucinol derivatization. Despite the clear evidence of successful transformation and presence of functional PsLAR, no complementation of the seed coat color or presence of DMACA reactive products were observed in the *anr* mutant background, nor was the presence of *trans-*flavan-*3*-ol and its derivatives observed in wild-type *Arabidopsis* (Additional file 8: Figure S6).

## Discussion

### Proanthocyanidin biosynthesis in pea (*Pisum sativum*) seeds

To investigate the PA diversity of pea seeds, the PA profiles of four pea cultivars were analyzed, and significant quantitative and qualitative variations in PA chemistry were observed. Of the cultivars examined, ‘Canstar’ lacked detectable PAs, while ‘Courier’, ‘Solido’, and ‘LAN3017’ had different quantities and/or types of PAs (Table 1; Figure 3). All three PA-containing cultivars contained PA levels comparable to that found in blueberries, cranberries, sorghum (high tannin whole grain extrudate) and hazelnuts [40]. ‘Courier’ and ‘Solido’ PAs are composed primarily of prodelphinidin subunits (tri-hydroxylated B-ring; Figure 1; Table 1), similar to the pea cultivars ‘CDC Acer’ and ‘CDC Rocket’ [41], and those found in tea [42]. In contrast, ‘LAN3017’, was composed of procyanidin-type subunits (di-hydroxylated B-ring; Figure 1; Table 1). These differences in PA subunit composition may impact the nutritional quality as tri-hydroxylated flavan-*3*-ols (e.g. GC and EGC) have a higher antioxidant potential than di-hydroxylated forms (e.g. CT and EC) [43]. Additionally, the mean degree of polymerization of ‘LAN3017’ PAs are 2–3 fold higher than ‘Courier’ or ‘Solido’ PAs. The mechanism controlling PA polymerization remains unknown, but it is particularly relevant as bioavailability after consumption by animals and humans is inversely related to polymer length [4]. In this regard, pea offers a valuable system to investigate the molecular basis for subtle biochemical differences in PA biosynthesis, and the pea cultivars with different PA profiles can be integrated into animal and human nutritional studies.

Although delphinidin-derived *epi*-gallocatechin is the most abundant *cis*-flavan-3-ol subunit in the PAs of ‘Courier’ (Table 1), the PA precurors substrates, pelargonidin and cyanidin, were utilized more efficiently than delphinidin by recombinant PsANR in our *in vitro* assays (Table 3). In comparison, PAs of tea tree (*Camellia sinensis*) also contain a high proportion of delphinidin-derived flavan-*3*-ol subunits, and its ANR displayed a higher preference for delphinidin than the other two substrates, displaying consistent *in vivo* and *in vitro* substrate preference [33]. The discrepancy of substrate preference found in pea ‘Courer’ is enigmatic, but obviously substrate availability for PsANR dictates PA monomer types in pea. It is well established that relative expression of *F3’H* and *F3’5’H* determines the PsANR substrate availability. We speculate that a dominant expression of *F3’5’H* may have recently occurred in ‘Courier’, but downstream PsANR has not fully adapted to delphinidin as a preferred substrate in this cultivar.

### Contribution of *LAR* to proanthocyanidin biosynthesis in pea and other plants

With the discoveries of *ANR* (or *BANYULS*) from *Arabidopsis* [12] and *LAR* from *D. uncinatum* [13], it has been well accepted that *cis*-flavan-*3*-ols are synthesized by the consecutive reactions of ANS and ANR, and *trans*-flavan-*3*-ols are synthesized by LAR, from the common substrates flavan-3,4-diols (leucoanthocyanidins) (Figure 1). For LAR activity, biochemical data using purified recombinant LAR enzyme from this work and others (*D. uncinatum* [13], grape [39], and tea [33]) have shown that LAR efficiently catalyzes the synthesis of *trans*-flavan-*3*-ols from leucoanthocyanidins (e.g., leucocyanidin). In the DFR/LAR coupled assays shown here, the synthesis rates of the LAR products, catechin and gallocatechin, from their respective substrates (i.e., DFR substrate) were slightly lower than, but still comparable to, those of *cis*-flavan-*3*-ols by ANR. These data are consistent with the pea PA monomer profile composed of comparable amounts of *trans-* and *cis*-3-flavan-*3*-ols. Although *LAR* has been isolated from several plants, LAR kinetic data is scarce, due to the instability and inaccessibility of its substrates. The only *K*_*m*_ values reported are 5–26 μM for three types of leucoanthocyanidins from native *D. uncinatum* LAR [13]. In DFR/LAR coupled assays, the amount of LAR substrates (i.e. intermediates in coupled reaction) is expected to be very low. Thus, the efficient synthesis of PsLAR products observed in the coupled assays implies rapid consumption of low abundant intermediates by PsLAR and may reflect high affinity of the substrates to PsLAR.

The biochemical data for PsLAR strongly support its role in production of *trans*-flavan-*3*-ols in pea. However, data from *LAR* overexpression studies in heterologous plants suggest that production of *trans*-flavan-*3*-ols through LAR *in planta* may require more than the presence of enzymatically competent LAR protein. Previously, expression of *D. uncinatum* and *M. truncatula LAR* by constitutive viral promoters in two *LAR*-lacking (thus, *trans*-flavan-*3*-ol-free) plants, white clover (*Trifolium repens*) and tobacco (*Nicotiana tabaccum*), did not lead to the production of *trans*-flavan-*3*-ols [13,26]. In the present study, instead of using a constitutive promoter, the *Arabidopsis ANR* promoter (known to drive strong gene expression in the seed coat) was used to express *PsLAR* in the *Arabidopsis* seed coat [27]. We hypothesized that the *ANR* promoter will express *PsLAR* at appropriate developmental stages in seed coat cells, where substrates for PsLAR are abundant. Accordingly, *PsLAR* transcript, protein, and catalytic activity were clearly detected from the transgenic *Arabidopsis* siliques (Additional file 7: Figure S5); nonetheless, no restoration of seed coat PA phenotype was observed in *anr* mutant *Arabidopsis*, and no catechin PA extension or terminal subunits could be detected after phloroglucinolysis analysis from *LAR*-overexpressing wild-type *Arabidopsis* (Additional file 8: Figure S6). This result suggests that simply placing active PsLAR enzyme in the seed coat could not sufficiently redirect the metabolic flux toward *trans*-flavan-*3*-ols. With our data in mind, it is noteworthy that a protein complex channelling dihydromyricetin (DFR substrate) to gallocatechin (LAR product) was purified from forage legume, *Onobrychis viciifolia* [44]. Therefore, although speculative, PsLAR may need to form an enzyme complex with PsDFR *in vivo* to fully draw a metabolic flux towards *trans*-flavan-*3*-ol synthesis. Further studies are required to test this model in pea or transgenic *Arabidopsis*.

Both *PsLAR* and *PsANR* transcript abundance was high earlier in pea seed coat development (Figure 4B and C). *PsLAR* showed the highest expression at 6 DAA and its transcripts were rapidly reduced to a basal level by 10 DAA. *PsANR* displayed a wider range of expression with substantial transcript levels until 15 DAA. By 12 DAA, PA accumulation in the seed coat was approximately half-maximal, reaching maximal levels at 20 DAA (Figure 4A). Consistent with this result, the 454-sequencing read number of *PsLAR* from the 10–25 DAA seed coat samples was 17-fold lower than that of *PsANR* (Figure 1). Curiously, the molar percent of GC extension units (LAR product) in ‘Courier’ seed coat PAs steadily increased from 12 DAA to 30 DAA, but *PsLAR* transcript abundance was minimal by 12 DAA (Table 2; Figure 4C). This apparent inconsistency between *PsLAR* expression and GC incorporation in pea seed coat PAs suggests that a pool of flavan-*3*-ols is made earlier in seed coat development, and this pool supplies substrates for PA polymerization throughout the remainder of tissue development. Alternatively, PsLAR protein may have an unusually long half-life. An understanding of the mechanism of PA polymerization is required to better address this discrepancy. Intriguingly, it was reported that grape ANR can synthesize not only EC (*cis*-flavan-3-ol) but also CT (*trans*-flavan-*3*-ol) in 50:50 molar ratio by its intrinsic epimerase activity [32], and such epimerase activity was also recently observed in tea ANR [33]. However, epimerase activity for the conversion of *cis*-flavan-*3*-ols to *trans*-flavan-*3*-ols could not be found from PsANR in our study, suggesting the *trans*-flavan-*3*-ols in pea were not derived from PsANR epimerase activity.

Taking all data together from this work and others, LARs from different plants have displayed competent biochemical activity to transform leucoanthocyanidins to *trans*-flavan-*3*-ols *in vitro*; however, the lack of accumulation of *trans*-flavan-*3*-ols and their derivatives accompanying expression of *LAR* in heterologous *LAR*-free plants still raises questions. It appears that the analysis of a *LAR* knock-out in *trans*-flavan-*3*-ol abundant plants (e.g., pea, tea, and grape) is necessary to definitely establish *in vivo* function of *LAR*.

## Conclusions

In this report, our comprehensive chemical analyses of PAs from pea seed coat showed that pea PAs are composed of both *cis*- and *trans*-flavan-3-ols and that substantial quantitative and qualitative variations of PA subunits (e.g., degree of hydroxylation and polymerization) are present among different pea cultivars. The transcriptomics analysis of the PA-rich ‘Courier’ seed coat identified key biosynthetic genes for both *cis*- and *trans*-flavan-3-ol synthesis in agreement with the PA profile in pea. The catalytic identities of the two key genes for PA synthesis (*PsANR* and *PsLAR*) were further confirmed by biochemical assays. Despite potent *in vitro* activity of PsLAR, expression of *PsLAR* in Arabidopsis seed coat (both in wild-type and *ANR* knock-out backgrounds) was unable to redirect the metabolic flux towards *trans*-flavan-3-ol synthesis, implying a possible *in planta* metabolite channelling. We expect that the improved understanding of PA chemical variations and associated biosynthetic mechanisms will help us develop pea cultivars with desirable PA types and quantity.

## Methods

### Plant material and growth conditions

Mature air-dried seeds of the pea (*Pisum sativum* L.) cultivars, ‘Canstar’, ‘Courier’, ‘Solido’ and ‘LAN3017’ (grown in Lethbridge, Alberta, Canada in 2007 or in Barrhead or Namao, Alberta, Canada in 2008) were used for PA extraction or growth chamber studies. For growth chamber studies, seeds were planted at an approximate depth of 2.5 cm in 3-L plastic pots (3 seeds per pot) in Sunshine no. 4 potting mix (Sun Gro Horticulture, Vancouver, Canada) and sand at 4:1. Plants were grown in a climate-controlled growth chamber with a 16 h-light/8 h-dark photoperiod (19°/17°C) with an average photon flux density of 383.5 μE/m^2^/s (measured with a LI-188 photometer, Li-Cor Biosciences, Lincoln, Nebraska). Flowers were tagged at anthesis, and seeds were harvested at selected stages as identified by days after anthesis (DAA). Seeds were harvested directly onto ice at 6, 8, 10, 12, 15, 20, 25, and 30, DAA and dissected immediately into seed coats and embryos and then stored at −80°C.

### RNA isolation and cDNA preparation

For cloning and qRT-PCR assays, total RNA was isolated from ‘Courier’ pea seed coat tissue using a Qiagen RNeasy Plant Mini kit. Polyvinylpyrrolidone (PVP-40) was added to the extraction buffer at a final concentration of 2% (w/v) to reduce precipitation of RNA by phenolic compounds contained in the seed coats [45]. First-strand cDNA was synthesized using Superscript II reverse transcriptase and an Oligo-dT_12–18_ primer (Invitrogen). Synthesis was conducted according to the manufacturer’s protocol.

### Cloning of the pea flavonoid genes *ANR*, *DFR* and *LAR*

*PsANR* was cloned from cDNA prepared from 20 DAA ‘Courier’ seed coat tissue using degenerate primers (Additional file 9: Table S3) based on conserved amino acid regions of ANR in *Medicago truncatula* (AAN77735.1), *Malus x domestica* (AAZ79363.1), *Fragaria ananassa* (ABG76843.1), *Arabidopsis thaliana* (AAF23859.1) and *Vitis vinifera* (AAZ82409.1). PCR reactions (50 μL) were run for a total of thirty-five cycles using 1 unit of Phusion polymerase (New England Biolabs), GC buffer (New England Biolabs), 0.2 mM dNTPs and 2 pmol forward and reverse primers, and 250 ng of 20 DAA Courier seed coat cDNA. Amplified fragments were cloned into pBlueScript II SK (−) (Stratagene) and sequenced. The full-length sequence of *PsANR* was recovered by rapid amplification of cDNA ends (RACE) using a SMART RACE cDNA Amplification kit (Clontech). RACE fragments were ligated into a pGEM-T Easy vector (Promega) and sequenced. Full-length *PsANR* was cloned into the Gateway donor vector pDONR221 (Invitrogen). The complete ORF of *PsDFR* was retrieved from the Courier 454-pyrosequencing data. The *PsLAR* sequence in the Courier 454-sequencing data lacked the 5’-end; therefore, 5’-RACE was performed to obtain the complete transcript sequence. The 5’-RACE fragment was ligated into a pGEM-T Easy vector (Promega) and sequenced. Full-length *PsDFR* and *PsLAR* were cloned into pDONR221 (Invitrogen) vector according to the manufacturer’s protocol. See supplementary materials for primer sequences.

### Recombinant expression and purification of pea ANR, DFR and LAR

Using the Gateway vector system, *PsANR*, *PsDFR* and *PsLAR* were cloned into pDEST17 (Invitrogen), containing an N-terminal 6x histidine tag for purification, and then transformed into *E. coli* BL21-AI (Invitrogen). The bacteria were grown in LB ampicillin (100 μg mL^−1^) media in a shaker at 37°C to an OD_600_ of 0.4-0.6 at which point the cultures were transferred to a refrigerator shaker for an additional 20–30 minutes at 12°C (ANR) or 15°C (DFR and LAR). Expression was then induced by adding L-arabinose (Sigma) to a final concentration of 0.2% (w/v). Following overnight incubation at 12°C or 15°C, the bacteria were pelleted by centrifugation at 4°C at 8000× g for 20 min. Pellets were resuspended in 1% original culture volume in lysis/wash buffer 1 (100 mM Tris–HCl pH 8, 10 mM imidazole, 10% glycerol, 0.1% Triton X-100, 10 mM β-mercaptoethanol). Cells were lysed by sonication on ice using a Microson Ultrasonic Cell Disrupter XL (Misonix, Farmingdale, NY) at 6 Watts for 10 seconds, repeated 8–10 times. The lysate was centrifuged at 12,000× g at 4°C for 20 min. For PsANR and PsDFR purification, the supernatant was applied to a 1 mL Bio-Scale Mini Profinity IMAC column (BioRad) using a BioLogic DuoFlow (Biorad) fast protein liquid chromatography (FPLC) machine. The column was washed at 1 mL min^−1^ with 6 mL lysis/wash buffer 1 followed by 6 mL wash buffer 2 (100 mM Tris–HCl pH 8, 20 mM imidazole, 10% glycerol, 0.1% Triton X-100, 10 mM β-mercaptoethanol, 1 M KCl). Recombinant protein was eluted at 1 mL min^−1^ with 7.5 mL of elution buffer (100 mM Tris–HCl pH 8, 250 mM imidazole, 10% glycerol, 300 mM KCl). The eluent was concentrated using an Amicon Ultra-30 column (Millipore). Aliquots of concentrated protein were immediately frozen in liquid nitrogen and stored at −80°C. Stability issues were encountered with PsLAR, and the recombinant enzyme was purified using a different method. After sonication and centrifugation, the supernatant was mixed for 1 hr on ice with nickel-NTA resins (Bio-rad) previously equilibrated with buffer (100 mM Tris–HCl (pH 8), 10% glycerol, 0.1% Triton X-100, supplemented with a protease-inhibitor cocktail (Roche)) containing 20 mM imidazole. The mixture was then loaded onto a Poly-Prep chromatography column (Bio-rad) and washed with 10 mL of buffer containing 20 mM imidazole. The column was eluted with 2 mL of buffer containing increasing concentrations of imidazole (50 mM, 100 mM, 200 mM, and 500 mM). Purity was checked by SDS-PAGE.

### PsANR *in vitro* assays

Substrates used were pelargonidin, cyanidin, and delphinidin (all from Extrasynthase, Genay, France). Authentic standards used were (−)-epiafzelechin (MicroSource, Gaylordsville, Connecticut) and (−)-epicatechin and (−)-epigallocatechin (both from Extrasynthase). To determine the linear range of PsANR activity, 5–100 μg of purified concentrated protein was assayed in a final reaction volume of 250 μL. The assays were run in 100 mM Tris–HCl containing 20 mM NADPH and 100 μM cyanidin. Coumarin was added to a final concentration of 25 μM as an internal standard. The reactions were incubated at 30°C for 30 min and stopped by extracting twice with 500 μL ethyl acetate, vortexing for 1 min, and centrifuging for 1 min. The ethyl acetate was evaporated under a N_2_ stream. The organic fraction was resuspended in 50 μL of 50% methanol and analyzed by high performance liquid chromatography (HPLC; Waters 2795 Separations Module) using a Sunfire C18 3.5 μm 4.6×150 mm column (Waters) and a photodiode array detector scanning between 100–400 nm. Peak area was quantified at 280 nm (EC, EAZ) and 270 nm (EGC). For the cyanidin and pelargonidin assays, the column was eluted using a linear gradient consisting of solvent A (100% H_2_O) and solvent B (100% acetonitrile) at a flow rate of 1.2 mL min^−1^ as follows: 0–8 min 20-50% B, 8.5-10 min 100% B. The chromatography gradient for delphinidin assays was 0–17 min 5-50% B, 17.01-18.5 min 100% B. Optimum temperature for PsANR activity was determined at temperatures between 25–60°C. Incubation time linearity was determined at 40°C for 15, 30, 60, 90, 120 and 240 min. Three buffers were used to test a pH range from 4–8.5 (50 mM citrate/phosphate: pH 4, 5, 6, 7; 50 mM MES (2-(N-morpholino) ethanesulfonic acid): pH 5, 6, 6.5, 7; 100 mM Tris–HCl: pH 7, 7.5, 8, 8.5). Kinetics assays were carried out in triplicate in a total reaction volume of 250 μL containing 100 mM Tris–HCl pH 7, using 40 μg ANR, 20 mM NADPH, at 42°C for 20 minutes.

### Recombinant PsDFR-PsLAR coupled enzyme assays

Substrates used were dihydroquercetin (DHQ; Extrasynthase) and dihydromyricetin (DHM; Chromadex, USA). Authentic standards used were (+)-catechin and (−)-gallocatechin (both from Sigma). *In vitro* coupled enzyme activity assays were carried out using 100 mM Tris–HCl, 50 μg PsDFR, 25 μg PsLAR, 100 μM DHM, 2 mM NADPH at temperatures from 22°C to 68°C for 30 minutes to determine the optimum temperature for the coupled reaction. The optimum pH of the coupled assay was determined using 50 mM MES (pH 5, 6, 7), 100 mM sodium phosphate buffer (pH 5.5, 6, 7) or 100 mM Tris–HCl (pH 7, 7.5, 8, 8.5). 100 μM DHM, 50 μg PsDFR and 25 μg PsLAR were added to each 250 μL reaction. NADPH was added to a final concentration of 2 mM. HPLC analysis of ANR assay products were performed as described above. Acetosyringone was used as an internal standard.

### *Arabidopsis PsLAR* transgenic plants

pKGWFS7 [46], contained a seed coat specific expression cassette (P_ANR_::*FLAG*::*PsLAR*::T35S) generated by PCR-stitching (see Additional file 9: Table S3 for primers), consisting of a 1367 bp portion of the native *A. thaliana BAN* promoter, shown to be sufficient to drive seed coat specific expression of a GUS reporter [27]. Briefly, full-length *PsLAR* was cloned from ‘Courier’ 10 DAA cDNA using primers 15 and 16, the P_ANR_ fragment was cloned from *Arabidopsis* Columbia-0 genomic DNA using primers 20 and 21, and T35S was cloned from pKWG2D [46] using primers 18 and 19, which also introduced a 5’-overlap region with 3’-*PsLAR. PsLAR* and T35S were stitched together using primers 17 and 19, which also added a 5’-*FLAG* tag to *PsLAR*. A 3’overlap region with 5’-*FLAG*::*PsLAR* was added to the P_ANR_ fragment using primers 20 and 22. The P_ANR_::*FLAG* and *FLAG*::*PsLAR*::T35S constructs were stitched together using primers 19 and 23. PCR reactions (50 μL) were run for a total of thirty cycles using 1 unit of Phusion polymerase (New England Biolabs), HF buffer (New England Biolabs), 0.2 mM dNTPs and 0.4-0.5 pmol forward and reverse primers. Touchdown PCR was used for the stitching reactions with an initial five cycles at an annealing temperature (Tm) dependent on the overlap regions involved. The Tm of the subsequent twenty-five cycles was dependent on the PCR primer Tm. Additionally, when stitching, the templates were added in equal molar ratios and PCR primers were added after the initial five cycles.

The vector was transformed into *Agrobacterium tumefaciens* GV3101. *Arabidopsis* Columbia-0 and an ANR T-DNA insertion line (SALK_040250C; ANR knock out line) were transformed by floral tip [47], T1 seeds were tested for kanamycin resistance and positive transformants were confirmed by genomic PCR using primers 16 and 20.

Activity of PsLAR in the transgenic lines was confirmed by *in vitro* assays using crude protein extracts. Immature siliques (40 mg) were ground first in liquid nitrogen and then in 600 μL of ice-cold extraction buffer (50 mM Tris–HCl (pH 7), 1% (w/v) PVP (polyvinylpyrrolidone), 0.2 mM PMSF (phenylmethylsulfonyl fluoride), 10% glycerol, 5 mM sodium metabisulfite, and 1 mM 2-mercaptoethanol). Following grinding, the mixture was centrifuged at 11,000× g for 10 min at 4°C. 150 μg total soluble protein with or without 130 μg of recombinant PsDFR was incubated in 50 mM MES (pH 6) with 2 mM NADPH and 100 μM dihydroquercetin in a final volume of 500 μL. The reaction was incubated at 35°C for a total of 90 min. Silique total soluble protein was added 30 min after the start of assay. For LC-MS analysis, the reactions were stopped and extracted as described above.

### Liquid chromatography mass spectrometry (LC-MS/MS)

PsANR reaction products were confirmed using an Agilent Technologies (Santa Clara, California) 6410 Triple Quad LC-MS/MS with a 1200 Series liquid chromatography system equipped with an electron spray ionization source and an Eclipse Plus C18 1.8 μm 2.1×50 mm column (Agilent). Samples were extracted and prepared as described above. Products and standards were detected in positive ion mode using product ion scan. Mass to charge ratio (*m/z*) selected for fragmentation were: 291 for EC/CT, 307 EGC/GC and 275 for EAZ. Fragmentor energies were: EC, 85 V; EGC, 50 V; EAZ, 80 V. Collision energies were: EC/CT, 12 and 20 eV; EGC/GC, 0 and 20 eV; EAZ, 20 eV. Liquid chromatography solvents were A) 1% (v/v) aqueous acetic acid and B) acetonitrile. Gradients for the samples were: EAZ, 10-50% B 0–8 minutes, 50-100% B 8–10 minutes; EC/CT, 5% B 0–0.5 minutes, 5-30% B 0.5-8 minutes, 100% B 8–11 minutes; EGC/GC 30-100% B 0–8 min. Flow rates for all samples were 0.4 mL min^−1^.

The products from *in vitro* assays using total soluble *Arabidopsis* silique protein were run on a longer gradient to ensure adequate separation between catechin and epicatechin; 0-40% B 0–15 min, 40-100% B 15–15.5 min, using 0.2% acetic acid in 5% acetonitrile (solvent A) and 0.2% acetic acid in 95% acetonitrile (solvent B).

### Real-time quantitative PCR

Total RNA was extracted from 6–20 DAA pea seed coat tissue or immature *Arabidopsis* siliques as described above. Quantitative real-time PCR (qRT-PCR) was run on an Applied Biosystems StepOne machine using Power SYBR Green PCR mix (Applied Biosystems). A master mix containing SYBR Green and cDNA was prepared according to the manufacturer instructions and split evenly into plate wells containing 0.3 pmol of gene forward and reverse primers such that each well contained 1–3 ng of cDNA (from mRNA) or 5–25 ng of cDNA (from total RNA). Relative transcript abundance was determined using the ∆∆C_T_ analysis method using Actin as the reference gene and three to four technical replicates [48].

### Extraction, purification and identification of proanthocyanidins

To estimate seed coat total extractable PA concentration, seed coat tissue was lyophilized and ground to a fine powder using a Retsch ZM 200 mill (PA, USA) fitted with a 0.5 mm screen filter. For each sample, approximately 25 mg of processed seed coat tissue was weighed into a 15 mL Falcon tube. The samples were extracted with 10 ml of 80% methanol for 24 hr with shaking. After vortexing the slurry and centrifuging for 5 min at 4000 rpm, the supernatants were used for PA analysis as previously described [31]. In brief, 2 mL of the butanol:HCl reagent and 66.75 μL of iron reagent were added into a 15 mL glass culture tube. Then, 0.5 mL of clear sample extract was added to the tube and the mixture was vortexed. Two 350 μL aliquots of the above solution were removed for use as sample blanks, and the remaining solution was placed into a 95°C water bath. After 40 min at 95°C, the solution was allowed to cool at room temperature for 30 min. The reaction products, sample blanks, and a PA standard curve dilution series were monitored for absorbance at 550 nm using a 96 well UV plate reader (Spectra Max 190, Molecular Devices, CA, USA). The PA standard solution used was an extract from ‘CDC Acer’ pea seed coats purified as described previously [41]. PA subunit composition and degree of polymerization were characterized and quantified using a method of acid-catalyzed cleavage of the PAs followed by phloroglucinol derivatization (phloroglucinolysis), as described by Jin et al. [41]. Identification of the PA subunits was confirmed by LC-MS/MS also using the method of Jin et al. [41].

### PA localization in pea seed coats

Fresh seed coat tissues of ‘Courier’ (10, 12, 15, 20, 25, 30, and 35 DAA) located adjacent to the mid-region of the cotyledons were dissected into 1 × 3 mm cross sections and immediately immerged into a fixing solution as described by Van Dongen et al. [49]. Briefly, the fixing solution consisted of 2.9% paraformaldehyde, 0.2% glutaraldehyde, 2 mM calcium chloride (CaCl_2_), 10 mM sucrose, and 25 mM piperazine-N,N′-bis(2-ethanesulfonic acid) (PIPES). The pH of the fixing solution was adjusted to 7.5 using sodium hydroxide. After five days of fixing solution infiltration under vacuum at room temperature, the tissues were rinsed three times with 25 mM aqueous PIPES buffer and dehydrated using a graded ethanol series of 30% and 50% ethanol in 25 mM PIPES buffer (pH 7.5; v/v), followed by 70%, 96%, and 100% ethanol in water for 15 min each. After two more changes in 100% ethanol followed by two changes in propylene oxide, the tissues were then submerged into 1:1 Spurr’s resin and propylene oxide mixture for 2 hours. Then, the tissues were properly oriented into Spurr’s resin bath and cured at 60°C for 3 days. Tissue sections were sliced into 4 μm-thick sections using a Reichert Jung Ultracut E ultra-microtome (Scotia, NY, USA), affixed onto clean slides, and placed at 60°C until dry. For PA localization in developing pea seed coats, a celloidin coating was applied to all slides prior to the staining process to improve the adherence of tissue sections to the glass slides. Briefly, the slides were submerged into absolute ethanol for 10 seconds, then coated with celloidin solution (0.5% celloidin in 1:1 ethanol: ethyl ether) for 5 min, and rinsed with 70% ethanol. Tissue sections were then stained with 0.1% 4-dimethylamino-cinnamaldehyde (DMACA) solution at 60°C for 30 min. The slides were washed with 100% ethanol and dehydrated with two changes of toluene for 5 min each. Cover slides were placed on slide-mounted tissue sections using DPX mounting media (BDH Chemicals). Tissue sections were observed using a Zeiss AXIO scope A1 light microscope (Zeiss, Germany) and micrographs were taken with a microscope-mounted Optronics camera (Optronics, CA, USA) controlled by Picture Frame™ Application 2.3 software.

### 454-Pyrosequencing

Total RNA was isolated from 10 to 25 DAA ‘Courier’ seed coats (4 to 5 g) using an adopted CTAB (hexadecyltrimethylammonium bromide) buffer extraction method [50]. mRNA was purified from the total RNA preparation using an Oligotex mRNA Mini kit (Qiagen). Double stranded cDNA was synthesized from the mRNA according to the Joint Genome Institutes (US Department of Energy) cDNA Library Creation 454 Protocol (my.jgi.doe.gov/general/protocols/) and quantified using the Quant-iT PicoGreen dsDNA assay (Invitrogen). Approximately 2 μg of cDNA was sent to the National Research Council Plant Biotechnology Institute (NRC-PBI, Saskatoon, Canada) for sequencing using a Roche 454 Titanium pyrosequencer. Transcriptome assembly was performed by personnel at the National Research Council Plant Biotechnology Institute (NRC-PBI; Saskatoon, Canada) using GS De Novo Assembler version 2.6 (Roche, Branford, Connecticut).

### cDNA sequence deposition

The sequences of cDNAs described in this work were deposited in the GenBank data library under the following accession number: *PsANR*, KF516483; *PsDFR*, KF516484; *PsLAR*, KF516485.

## Additional files

Additional file 1: Table S1. Characterization of phloroglucinolysis products from pea seeds using LC-MS-MS analysis. Additional file 2: Figure S1. MS/MS patterns of ANR- and DFR/LAR-products. A, MS/MS data for ANR-products and *cis*-flavan-3-ol standards are shown. B, MS/MS data for DFR/LAR-products and *tran*-flavan-3-ol standards are shown. Additional file 3: Figure S2. Incubation of recombinant PsANR with epicatechin. A, (−)-epicatechin standard (retention time; RT, 6.30 min). B, (+)-catechin standard (RT, 4.85 min). C, PsANR incubated with epicatechin (RT, 6.26 min). Additional file 4: Table S2. Top 20 unigenes in ‘Courier’ 10–25 DAA seed coat transcriptome. Following assembly and UniProt annotation, contigs were sorted based on the number of reads comprising each contig. Additional file 5: Figure S3. Alignment of LAR protein sequences. *Pisum sativum* (Ps; KF516485), *Medicago truncatula* (Mt; XP_003591830.1), *Lotus corniculatus* (Lc; LAR2-1, ABC71328.1; LAR2-2, ABC71331.1), *Desmodium uncinatum* (Du; Q84V83.1), *Phaseolus coccineus* (Pc; CAI56322.1), *Vitis vinifera* (Vv; CAI26309.1). LAR characteristic amino acid motifs RFLP, ICCN, and THD marked by black bars above the *Pisum sativum* sequence. Additional file 6: Figure S4. Expression of PsANR, PsLAR, and PsDFR in *E. coli* and purification of their recombinant proteins. A) Recombinant PsANR. Crude extract (lane 1), washes 1 and 2 (lanes 2 and 3, respectively) and elutent (lane 4). B) Recombinant PsDFR and PsLAR. Lanes 1, 2 and 3 represent crude soluble protein, crude insoluble protein and purified concentrated protein, respectively, in PsDFR expressing culture. Lanes 4, 5 and 6 represent the similar samples from PsLAR expressing culture. Each lane contains approximately 12–15 μg of protein visualized by Coomassie staining. Additional file 7: Figure S5. RT-PCR and immunoblot analyses of PsLAR transcript and protein in *Arabidopsis* wild-type and *Arabidopsis ANR* knock-out lines. A) RT-PCR of *PsLAR* transcript (trans-transcript, above) and *AtDFR* (native transcript, below). Col: Arabidopsis Columbia; ANR KO: Arabidopsis ANR knock-out. Col GFP indicates *GFP*-expressing Arabidopsis Columbia line, which was used as a negative control. B) Immonoblot analysis of FLAG-tagged PsLAR (above) and Coumassie stained SDS-PAGE gels (below). C) ESI-LC-MS [M + H]^+^ (*m/z* = 291) extracted ion chromatographs from authentic (+)-catechin (left) compared to *in vitro* assays using crude total soluble protein extracted from *PsLAR* transgenic *Arabidopsis* siliques supplemented with (blue) or without (red) recombinant PsDFR and a vector control line with PsDFR (black). Additional file 8: Figure S6. Analysis of seed coat color and PA chemical profile from transgenic plants. A) Dried seeds (above) and p-dimethylaminocinnamaldehyde (DMACA) stained seeds (below) from GFP vector control line and ANR KO-PsLAR transgenic lines show a lack of proanthocyanidins or flavan-*3*-ols in the seed coat. B) HPLC chromatograms of the phloroglucinol acid hydrolysis products of proanthocyanidins extracted from the mature seeds of Col *PsLAR* 5 transgenic lines. No catechin-phloroglucinol (PA extension units; solid arrow) or catechin (PA terminal units; dashed arrow) were detected in Col LAR5. ‘LAN3017’ seed coat proanthocyanidins were used as a control for catechin-phloroglucinol. Peak 1, catechin-phloroglucinol; Peak 2, epicatechin-phloroglucinol; Peak 3 and peak 4 are (+)-catechin and (−)-epicatechin standards, respectively. Additional file 9: Table S3. List of PCR primers used in this study.

## Abbreviations

PA: Proanthocyanidin
mDP: Mean degree of polymerization
DAA: Days after anthesis
EC: Epicatechin
EAZ: Epiafzelechin
EGC: Epigallocatechin
DHM: Dihydromyricetin
DHQ: Dihydroquercetin
CT: Catechin
GC: Gallocatechin
AZ: Afzelechin
DMACA: *p*-dimethyl aminocinnamaldehyde
EST: Expressed sequence tag
