# Supplementary material for: Characterization of proanthocyanidin metabolism in pea (Pisum sativum) seeds
Source: BMC Plant Biol. 2014 Sep 16;14:238. doi: 10.1186/s12870-014-0238-y (PMC4175280; doi:10.1186/s12870-014-0238-y)
Supplement: Additional file 1: Table S1. — Characterization of phloroglucinolysis products from pea seeds using LC-MS-MS analysis. [file 12870_2014_238_MOESM1_ESM.pdf]

Table S1: Characterization of phloroglucinolysis products from pea seeds using LC-MS-MS analysis.

Compound[a]	Cultivar	t_R (min)[b]	$[M-H]^-$ [c]	Fragment ions
GC-P	'Solido'	7.4	429	303, 261, 177
EGC-P	'Solido'	9.6	429	303, 261, 177
GC	'Solido'	16.8	305	219, 137
GC Standard		18.1	305	231, 219, 179
CT-P isomer	'LAN3017'	17.2	413	287, 261, 161, 135
CT-P	'LAN3017'	21.3	413	287, 261, 217, 175
EC-P	'LAN3017'	22.1	413	287, 261, 175
EGC	'Solido'	33.4	305	219, 137
EGC Standard		34.4	305	219, 179, 137
CT	'LAN3017'	33.3	289	245, 173, 137
CT Standard		33.2	289	245, 205, 137
EC	'LAN3017'	44.3	289	245, 137

[a] GC-P, gallocatechin-(4α→2)-phloroglucinol; EGC-P, epigallocatechin-(4β→2)-phloroglucinol; EC-P, epicatechin-(4β→2)-phloroglucinol; CT-P, catechin-(4α→2)-phloroglucinol; CT, catechin; GC, gallocatechin; EGC, epigallocatechin; EC, epicatechin.

[b] Retention time on LC-MS. The gallocatechin and epigallocatechin standards were run at different times with the legume samples leading to some variation in retention time between the same compounds in the standards and samples.

[b] MS was run in the negative mode and all the molecular ions are $[M-H]^-$.
