# Supplementary figures and images for: Characterization of proanthocyanidin metabolism in pea (Pisum sativum) seeds
Source: BMC Plant Biol. 2014 Sep 16;14:238. doi: 10.1186/s12870-014-0238-y (PMC4175280; doi:10.1186/s12870-014-0238-y)

**A**

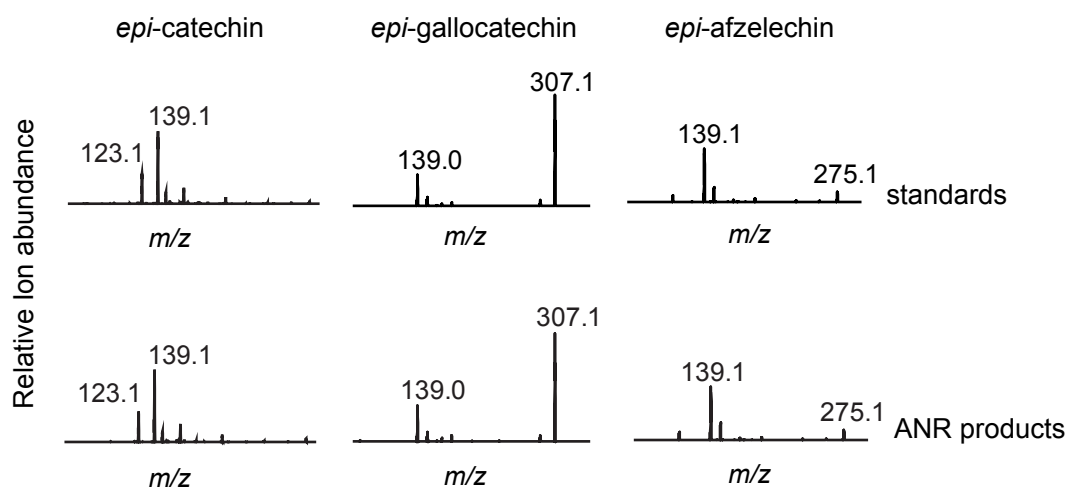

**B**

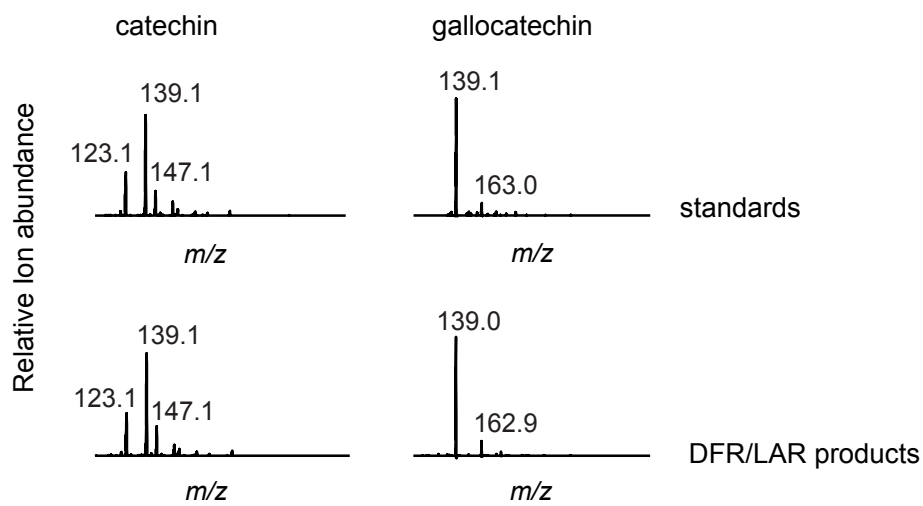

Supplement: Additional file 2: Figure S1. — MS/MS patterns of ANR- and DFR/LAR-products. A, MS/MS data for ANR-products and cis-flavan-3-ol standards are shown. B, MS/MS data for DFR/LAR-products and tran-flavan-3-ol standards are shown. [file 12870_2014_238_MOESM2_ESM.pdf]

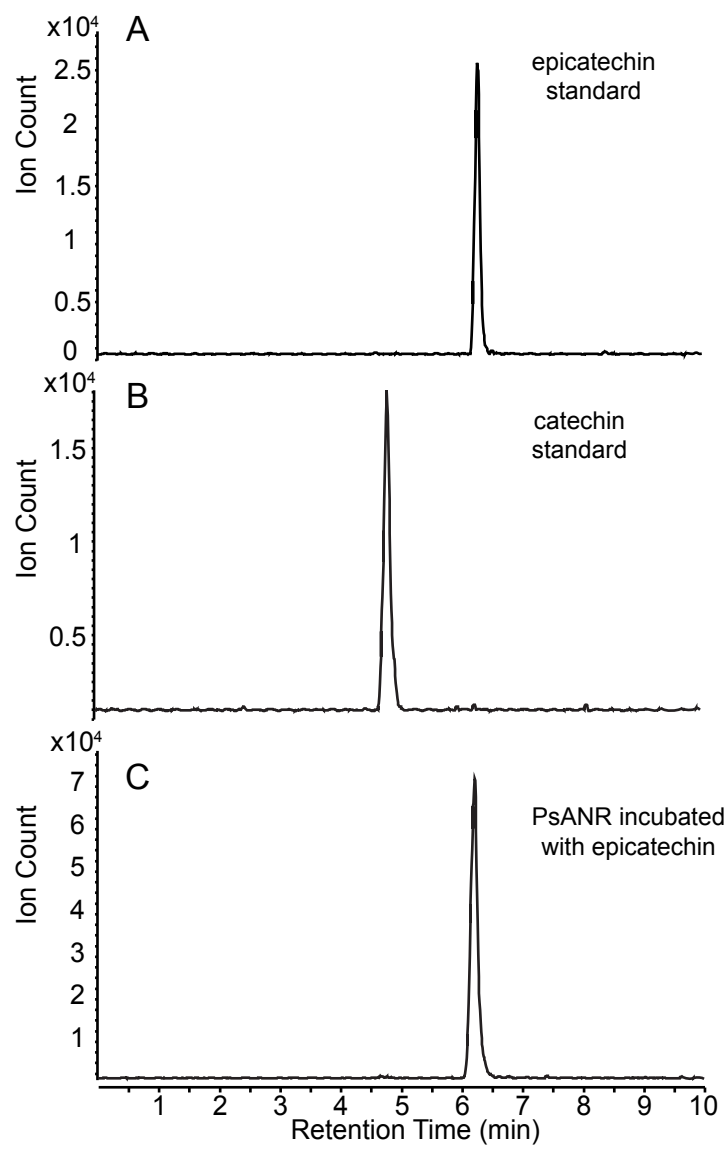

Supplement: Additional file 3: Figure S2. — Incubation of recombinant PsANR with epicatechin. A, (−)-epicatechin standard (retention time; RT, 6.30 min). B, (+)-catechin standard (RT, 4.85 min). C, PsANR incubated with epicatechin (RT, 6.26 min). [file 12870_2014_238_MOESM3_ESM.pdf]

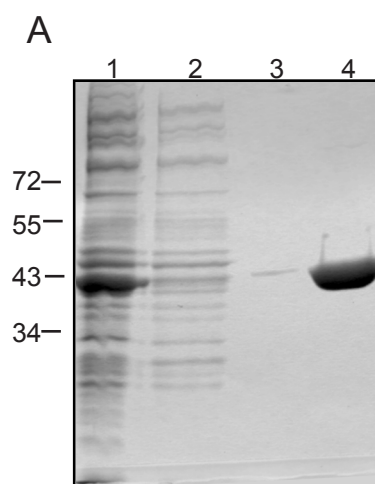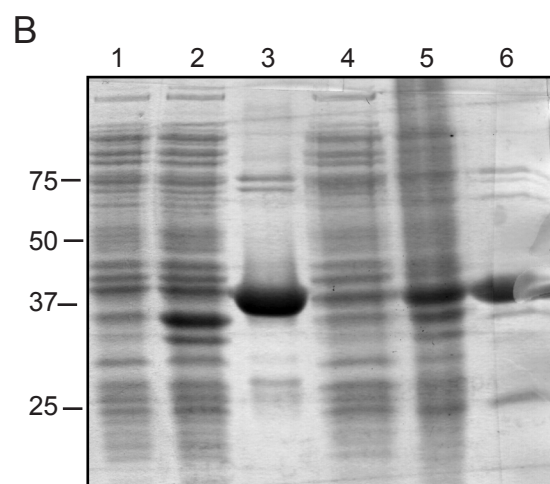

Supplement: Additional file 6: Figure S4. — Expression of PsANR, PsLAR, and PsDFR in E. coli and purification of their recombinant proteins. A) Recombinant PsANR. Crude extract (lane 1), washes 1 and 2 (lanes 2 and 3, respectively) and elutent (lane 4). B) Recombinant PsDFR and PsLAR. Lanes 1, 2 and 3 represent crude soluble protein, crude insoluble protein and purified concentrated protein, respectively, in PsDFR expressing culture. Lanes 4, 5 and 6 represent the similar samples from PsLAR expressing culture. Each lane contains approximately 12–15 μg of protein visualized by Coomassie staining. [file 12870_2014_238_MOESM6_ESM.pdf]

A

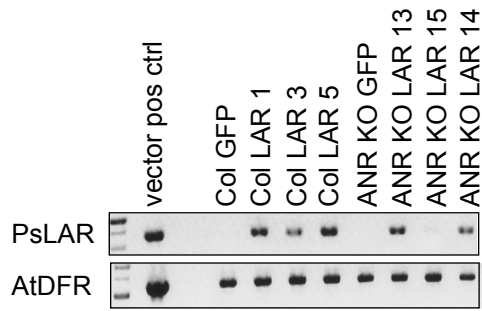

B

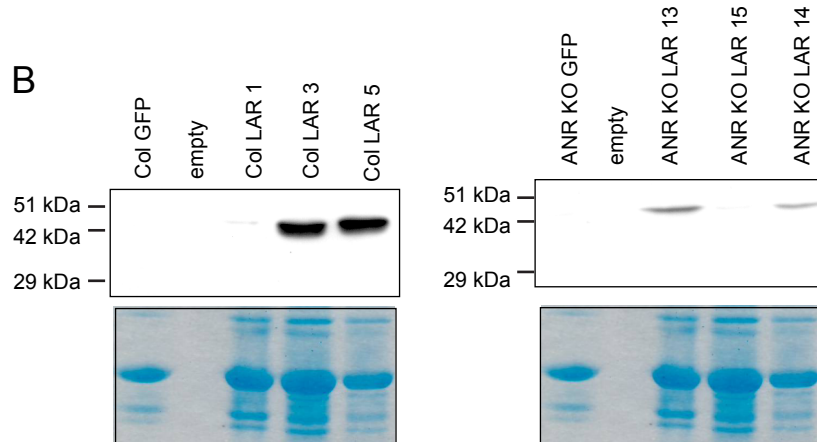

C

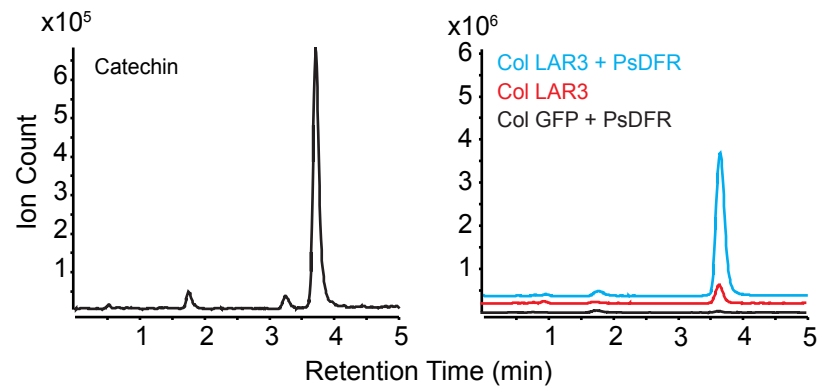

Supplement: Additional file 7: Figure S5. — RT-PCR and immunoblot analyses of PsLAR transcript and protein in Arabidopsis wild-type and Arabidopsis ANR knock-out lines. A) RT-PCR of PsLAR transcript (trans-transcript, above) and AtDFR (native transcript, below). Col: Arabidopsis Columbia; ANR KO: Arabidopsis ANR knock-out. Col GFP indicates GFP-expressing Arabidopsis Columbia line, which was used as a negative control. B) Immonoblot analysis of FLAG-tagged PsLAR (above) and Coumassie stained SDS-PAGE gels (below). C) ESI-LC-MS [M + H]+ (m/z = 291) extracted ion chromatographs from authentic (+)-catechin (left) compared to in vitro assays using crude total soluble protein extracted from PsLAR transgenic Arabidopsis siliques supplemented with (blue) or without (red) recombinant PsDFR and a vector control line with PsDFR (black). [file 12870_2014_238_MOESM7_ESM.pdf]

A

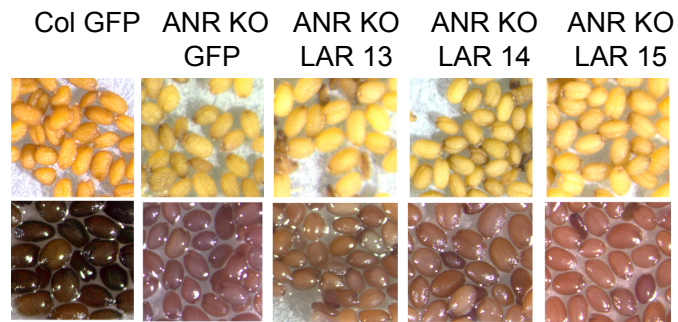

B

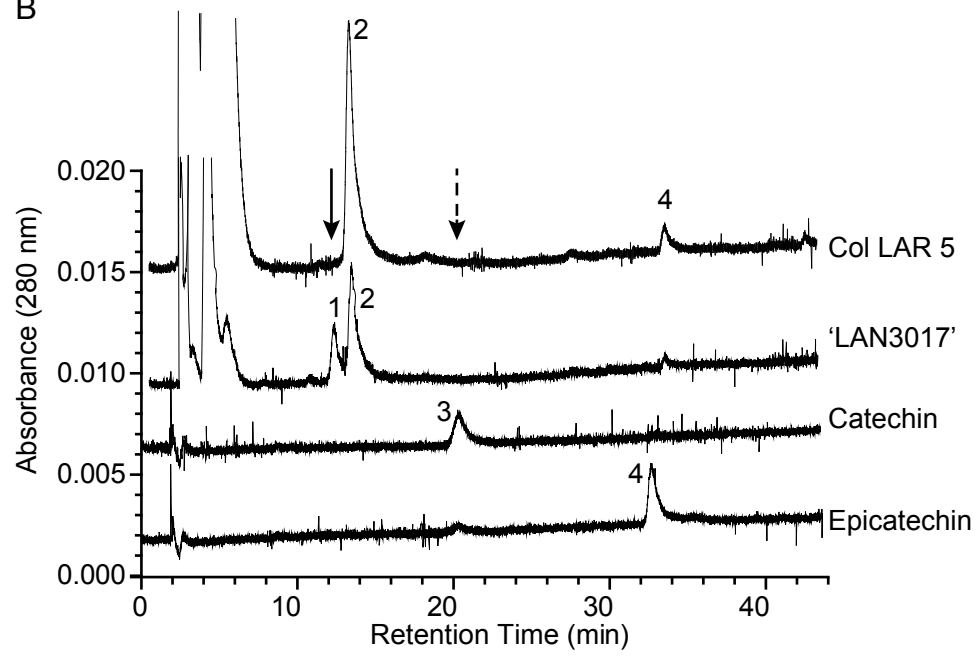

Supplement: Additional file 8: Figure S6. — Analysis of seed coat color and PA chemical profile from transgenic plants. A) Dried seeds (above) and p-dimethylaminocinnamaldehyde (DMACA) stained seeds (below) from GFP vector control line and ANR KO-PsLAR transgenic lines show a lack of proanthocyanidins or flavan-3-ols in the seed coat. B) HPLC chromatograms of the phloroglucinol acid hydrolysis products of proanthocyanidins extracted from the mature seeds of Col PsLAR 5 transgenic lines. No catechin-phloroglucinol (PA extension units; solid arrow) or catechin (PA terminal units; dashed arrow) were detected in Col LAR5. ‘LAN3017’ seed coat proanthocyanidins were used as a control for catechin-phloroglucinol. Peak 1, catechin-phloroglucinol; Peak 2, epicatechin-phloroglucinol; Peak 3 and peak 4 are (+)-catechin and (−)-epicatechin standards, respectively. [file 12870_2014_238_MOESM8_ESM.pdf]
