# Supplementary material for: Characterization of proanthocyanidin metabolism in pea (Pisum sativum) seeds
Source: BMC Plant Biol. 2014 Sep 16;14:238. doi: 10.1186/s12870-014-0238-y (PMC4175280; doi:10.1186/s12870-014-0238-y)
Supplement: Additional file 4: Table S2. — Top 20 unigenes in ‘Courier’ 10–25 DAA seed coat transcriptome. Following assembly and UniProt annotation, contigs were sorted based on the number of reads comprising each contig. [file 12870_2014_238_MOESM4_ESM.pdf]

Table S2. Top 20 unigenes in ‘Courier’ 10-25 DAA seed coat transcriptome. Following assembly and UniProt annotation, contigs were sorted based on the number of reads comprising each contig.

Sequence ID	Read number	E-value	Hit ID-UniProt	Functional description
CL1Contig1039	218	1.0e-173	Q9FT05_CICAR	Cationic peroxidase
CL1Contig946	217	1.0e-141	ACCO_PEA	1-aminocyclopropane-1-carboxylate oxidase
CL1Contig433	216	0	A2Q639_MEDTR	Indole-3-acetic acid-amido synthetase
CL1Contig362	195	0	C3TS12_CICAR	Methionine synthase
CL1Contig1430	187	1.0e-157	Q84XT1_MEDTR	**Anthocyanidin reductase**
CL1Contig1488	186	0	A5Y5L3_SOYBN	**Flavonoid 3',5' hydroxylase**
CL1Contig753	165	1.0e-130	Q8RU51_ORYSJ	Putative Glucan 1,3-beta-glucosidase
CL1Contig1170	143	0	AMYB_VIGUN	1,4-alpha-D-glucan maltohydrolase
CL1Contig898	125	4.0e-19	Q8VYY0_PEA	Dehydrin-related protein
CL1Contig383	124	0	VPE_VICSA	Vacuolar-processing enzyme, Proteinase B
CL1Contig1280	115	0	Q2HRK7_MEDTR	Protease-associated protein
CL1Contig610	105	7.0e-52	Q9MB25_VIGUN	Pathogenesis-related protein
CL1Contig12	100	3.0e-45	Q0GPF8_SOYBN	BZIP transcription factor bZIP124
CL1Contig1745	94	4.0e-64	B9SRU3_RICCO	Carboxylic ester hydrolase, putative
CL1Contig780	87	3.0e-71	P93332_MEDTR	Nodulin MtN3 family protein
CL1Contig1844	83	1.0e-135	G2OX1_PEA	Gibberellin 2-beta-dioxygenase 1
CL1Contig59	79	0	Q6UDA0_TRIPR	Actin
CL1Contig1264	78	1.0e-178	PRS7_ARATH	26S protease regulatory subunit 7
CL1Contig78	77	0	Q9FEU4_PEA	Putative serine carboxypeptidase
