# Supplementary material for: Characterization of proanthocyanidin metabolism in pea (Pisum sativum) seeds
Source: BMC Plant Biol. 2014 Sep 16;14:238. doi: 10.1186/s12870-014-0238-y (PMC4175280; doi:10.1186/s12870-014-0238-y)
Supplement: Additional file 5: Figure S3. — Alignment of LAR protein sequences. Pisum sativum (Ps; KF516485), Medicago truncatula (Mt; XP_003591830.1), Lotus corniculatus (Lc; LAR2-1, ABC71328.1; LAR2-2, ABC71331.1), Desmodium uncinatum (Du; Q84V83.1), Phaseolus coccineus (Pc; CAI56322.1), Vitis vinifera (Vv; CAI26309.1). LAR characteristic amino acid motifs RFLP, ICCN, and THD marked by black bars above the Pisum sativum sequence. [file 12870_2014_238_MOESM5_ESM.pdf]

```
Ps     MAPTSSPPTTLASKNRVLIIGATGFMGKFLTEASLSSSHPTYLLIRPGGPLLSPKSTTIK
Mt     MAPSSSP-TTPISKGRVLIVGATGFMGKFVTEASISTAHPTYLLIRPG-PLISSKAATIK
Lc2-1  ----------MATKGRVLIIGATGFMGRFMAEASLAAAHPTYLLVRQP--LIPSKATIVK
Lc2-2  ----------MATKGRVLVIGATGFMGRFMAEASLVTAHPTYLLVRQP--LIPSKATIVK
Du     --MTVSGAIPSMTKNRTLVVGGTGFIGQFITKASLGFGYPTFLLVRPG-PVSPSKAVIIK
Pc     --MVTSPTIPSHTKARVLIIGATGFIGKFVTEASLLTAHPTYLLLRPP-PLVPSKDAIVK
Vv1    --MTVSP--VPSPKGRVLIAGATGFIGQFVAAASLDAHRPTYILARPG-PRSPSKANIFK

Ps     TFQDKGAIIVYGVVDNKEFMEKILKKYEIDIVISAIG-AESLLDQITLVEAMKSIKTVKR
Mt     TFQEKGAIVIYGVVNNKEFVEMILKKYEIDTVISAIG-AESLLDQLTLVEAMKSIKTIKR
Lc2-1  TFQDKGAIVIQGVMNDKEFMQKILKEYQIDIVISTVGGAHGLLDQLTLVEAMKSVNTIKR
Lc2-2  TFQDKGAIVIQGVMNDKEFMQKILKEYQIDIVISTVGGAHGLLDQLTLVEAMKSVNTIKR
Du     TFQDKGAKVIYGVINDKECMEKILKEYEIDVVISLVG-GARLLDQLTLLEAIKSVKTIKR
Pc     TFQEKGAMIIHGVINNKDFVEKILKEHEIDIVISAIG-AKSLLDQLILVEAMKSLKTIKR
Vv1    ALEDKGAIIVYGLINEQEAMEKILKEHEIDIVVSTVG-GESILDQIALVKAMKAVGTIKR

Ps     FLPSEFGHDVDRADPVEPGLAMYKQKRLVRRVIEESGVPYTYICCNSIASWPYYNNCHPS
Mt     FLPSEFGHDVDRADPVEPGLAMYKQKRLVRRVIEESGVPYTYICCNSIASWPYYDNCHPS
Lc2-1  FLPSEFGHDVDRADPVEPGLAMYKEKRLVRRVIEESGIPYTYICCNSIASWPYYNNCHPS
Lc2-2  FLPSEFGHDVDRADPVEPGLAMYKEKRLVRRVIEESGIPYTYICCNSIASWPYYNNCHPS
Du     FLPSEFGHDVDRTDPVEPGLTMYKEKRLVRRAVEEYGIPFTNICCNSIASWPYYDNCHPS
Pc     FLASEFGHDVDRADPVEPGLTMYKEKQLVRRVVEQSGVPYTNICCNSIASWPYYDNCHPS
Vv1    FLPSEFGHDVNRADPVEPGLNMYREKRRVRQLVEESGIPFTYICCNSIASWPYYNNIHPS

Ps     SLPPPLDQLHVYGNGNVKAYFVDGFDIGKFTMKIVDDHRTINKNIHFRPSINCYSMNELA
Mt     QLPPPLDQLHIYGHGNVKAYFVDGYDIGKFTMKVVDDERTINKSVHFRPSTNCYSMNELA
Lc2-1  QLPPPLDQMHIYGDGTVKAYFVDGFDIGKFTMKLVDDARALNKVVHFRPSSNYYSMNELA
Lc2-2  QLPPPLDQMHIYGDGTVKAYFVDGFDIGKFTMKLVDDARALNKVVHFRPSSNYYSMNELA
Du     QVPPPMDQFQIYGDGNTKAYFIDGNDIGKFTMKTIDDIRTLNKNVHFRPSSNCYSINELA
Pc     QLPPPLDQLQIYGHGNVKAYFVDGIDIGKFTMKVIDDVKTINKNVHFRPSKNCYSINELA
Vv1    EVLPPTDFFQIYGDGNVKAYFVAGTDIGKFTMKTVDDVRTLNKSVHFRPSCNCLNINELA

Ps     SLWESKIARKIPRLFVSENDLLAIAAENIIPESVVASLTHDIFINGCQVSYKIDGVHDVE
Mt     SLWENKIARKIPRAIVSEDDLLGIAAENCIPESVVASITHDIFINGCQVNFKIDGIHDVE
Lc2-1  PLWENKVGRKIPRVTISEDDLLALAAENCIPESIVASITHDIFIKGCQVNYNIDGVHDIE
Lc2-2  SLWENKVGRKIPRVTISEDVLLALAAENCIPESIVASITHDIFIKGCQVNYNIDGVHDIE
Du     SLWEKKIGRTLPRFTVTADKLLAHAAENIIPESIVSSFTHDIFINGCQVNFSIDEHSDVE
Pc     SLGEMKIGRTIPRVTISEDDLLAAAAENCIPQSIVASFTHDIFIKGCQVNFSIDGVDDVE
Vv1    SVWEKKIGRTLPRVTVTEDDLLAAAGENIIPQSVVAAFTHDIFIKGCQVNFSIDGPEDVE

Ps     ISTLYPGESFRSMEDCFESFVVMAADKIRKGENG--------------------------
Mt     ISTLYPGESFRSLEDCFESFVAMAADKIHKGENG--------------------------
Lc2-1  IGTLYPDETFRSLEDCFEDFVTMIVEKIHKGENE--------------------------
Lc2-2  VGTLYPDETFRSLEDCFEDFVTMIVEKIHKGENE--------------------------
Du     IDTLYPDEKFRSLDDCYEDFVPMVHDKIHAGKSGEIKIKDGKPLVQTGTIEEINKDIKTL
Pc     ISTLYPDEEFRSLEDCYEDFAHMIEDNIHKGEHK--------------------------
Vv1    VTTLYPEDSFRTVEECFGEYIVKIEEKQPTADSA--------------------------

Ps     ---------VAGGTKSMVEPVIITASC-----
Mt     ---------VTGGTKALVEPVPITASC-----
Lc2-1  ---------VYG-TKSLVEAVPITASC-----
Lc2-2  ---------VYG-TKSLVEAVPITASC-----
Du     VETQPNEEIKKDMKALVEAVPISAMG-----
Pc     ---------ITG-TKSVVEAVPIMASCGNIYE
Vv1    ---------IAN-TGPVVGMRQVTATCA----
```
