# Supplementary material for: Characterization of proanthocyanidin metabolism in pea (Pisum sativum) seeds
Source: BMC Plant Biol. 2014 Sep 16;14:238. doi: 10.1186/s12870-014-0238-y (PMC4175280; doi:10.1186/s12870-014-0238-y)
Supplement: Additional file 9: Table S3. — List of PCR primers used in this study. [file 12870_2014_238_MOESM9_ESM.pdf]

Table S3. List of PCR primers used in this study.

No.	Primer names[a]	Sequence (5’ > 3’)
1	*PsANR* degenerate F	ATAGTCTAGAGAYATGATHAARCCNGC
2	*PsANR* degenerate R	CGATTCTAGAGCRCARCADATRTAYCT
3	*PsANR* RACE 5’-gsp	CGGCGATCAAAGGTGTGTTGAATGTGTTG
4	*PsANR* RACE 5’-nested	CTGGTCTGATGTTGAGTTTCTGAATGCGGC
5	*PsANR* RACE 3’-gsp	TGAGCTCGGCAAATATCCTCGACATGA
6	*PsANR* RACE 3’-nested	GTGGCAGAGAAAGAATCAGCTTCTGG
7	*PsANR* Gateway F	AAAAAGCAGGCTTAATGGCTAGTATCAAAGAAG
8	*PsANR* Gateway R	AGAAAGCTGGGTATTACTTCTTCAAGACCCCC
9	*PsLAR* RACE 5’-gsp	CTGATCCAACGGTGGAGGAAGC
10	*PsLAR* RACE 5’-nested	CCAGATTCCTCGATCACACGTCTAACC
11	*PsLAR* Gateway F	AAAAAGCAGGCTTCATGGCACCAACTTC ATCAC
12	*PsLAR* Gateway R	AGAAAGCTGGGTCTCAACAGGAAGCTGTGAT TATTAC
13	*PsDFR* Gateway F	AAAAAGCAGGCTTCATGGGTTCGGTGTCG
14	*PsDFR* Gateway R	AGAAAGCTGGGTCTTATTTCTTCATGGTGTCATTAACTTCGGTC
15	*PsLAR* F	ATGGCACCAACTTCATCACCACCAACCAC
16	*PsLAR* R	TCAACAGGAAGCTGTGATTATTACTGGTTCT ACC
17	5’ *FLAG* tag+*PsLAR* overlap F	ATGGATTACAAGGATGACGACGATAAGATCATGGCACCAACTTCATCACCACC
18	*T35S* overlap with 3'-LAR F	CCAGTAATAATCACAGCTTCCTGTTGACGGCCATGCTAGAGTCCGC
19	*T35S* Gateway R	AGAAAGCTGGGTCAGGTCACTGGATTTTGGT TTTAGG
20	*Ath* P_{ANR} F	CCAGGAGGTTTTCAAAGACTATGGAGTG
21	*Ath* P_{ANR} R	CATAACAACTAAATCTCTATCTCTGTAATTTCAAAAGTACAATC
22	*Ath* P_{ANR} *FLAG* overlap R	TTATCGTCGTCATCCTTGTAATCCATGATTGTACTTTTGAAATTACAGAGATAGAG
23	*Ath* P_{ANR} Gateway F	AAAAAGCAGGCTTCCCAGGAGGTTTTCAAAG ACTATGGA
24	*PsActin* qRT-PCR F	TTCTCACTGAAGCTCCGCTTAACC
25	*PsActin* qRT-PCR R	CAATACCAGTTGTACGGCCACTAGC
26	*PsANR* qRT-PCR F	TCAGAATACCTGTGTTCCCGAGCTTG
27	*PsANR* qRT-PCR R	CCTTGCGGCAATCCTCGAATTTAGT
28	*PsLAR* qRT-PCR F	TCCTGTGGAGCCAGGTTTAGCAAT
29	*PsLAR* qRT-PCR R	AGTAAGGCCAAGATGCGATGGAGT
30	*PsDFR* qRT-PCR F	CGTTCGCGATCCAGATAACGTGAA
31	*PsDFR* qRT-PCR R	ACCCTCTTCAGCAAGATCAGCCTT
32	*Ath ubiquitin* qRT-PCR F	GGCCTTGTATAATCCCTGATGAATAAG
33	*Ath ubiquitin* qRT-PCR R	AAAGAGATAACAGGAACGGAAACATA
34	*Ath ANR* qRT-PCR F	ACCGGGAAAGAAATGCATGTGACC
35	*Ath ANR* qRT-PCR R	ATGGGCACGACGTAAATCGTCTAC
36	*Ath ANS* qRT-PCR F	TGGGTTGGTGAATAAGGAGAAG
37	*Ath ANS* qRT-PCR R	GGCAACGGCTTAAGAACAATC
38	*Ath CHS* qRT-PCR F	TGACTGGAACTCCCTCTTCT
39	*Ath CHS* qRT-PCR R	GCCCTCATCTTCTCTTCCTTTAG
40	*Ath DFR* qRT-PCR F	GGTCGGTCCATTCATCACAA
41	*Ath DFR* qRT-PCR R	CGTTGCATAAGTCGTCCAAATG

[a] Forward and reverse primer are abbreviated as F and R; gsp, gene specific primer.
